# Metropolitan-scale heralded entanglement of solid-state qubits

**DOI:** 10.1126/sciadv.adp6442

**Published:** 2024-10-30

**Authors:** Arian J. Stolk, Kian L. van der Enden, Marie-Christine Slater, Ingmar te Raa-Derckx, Pieter Botma, Joris van Rantwijk, J. J. Benjamin Biemond, Ronald A. J. Hagen, Rodolf W. Herfst, Wouter D. Koek, Adrianus J. H. Meskers, René Vollmer, Erwin J. van Zwet, Matthew Markham, Andrew M. Edmonds, J. Fabian Geus, Florian Elsen, Bernd Jungbluth, Constantin Haefner, Christoph Tresp, Jürgen Stuhler, Stephan Ritter, Ronald Hanson

**Affiliations:** ^1^ QuTech and Kavli Institute of Nanoscience, Delft University of Technology, 2628 CJ, Delft, Netherlands.; ^2^ Netherlands Organisation for Applied Scientific Research (TNO), P.O. Box 155, 2600 AD, Delft, Netherlands.; ^3^ Element Six Innovation, Fermi Avenue, Harwell Oxford, Didcot, Oxfordshire OX11 0QR, UK.; ^4^ Fraunhofer Institute for Laser Technology ILT, 52074 Aachen, Germany.; ^5^ RWTH-Aachen University, 52074 Aachen, Germany.; ^6^ TOPTICA Photonics AG, Lochhamer Schlag 19, 82166 Graefelfing, Germany.

## Abstract

A key challenge toward future quantum internet technology is connecting quantum processors at metropolitan scale. Here, we report on heralded entanglement between two independently operated quantum network nodes separated by 10 kilometers. The two nodes hosting diamond spin qubits are linked with a midpoint station via 25 kilometers of deployed optical fiber. We minimize the effects of fiber photon loss by quantum frequency conversion of the qubit-native photons to the telecom L-band and by embedding the link in an extensible phase-stabilized architecture enabling the use of the loss-resilient single-click entangling protocol. By capitalizing on the full heralding capabilities of the network link in combination with real-time feedback logic on the long-lived qubits, we demonstrate the delivery of a predefined entangled state on the nodes irrespective of the heralding detection pattern. Addressing key scaling challenges and being compatible with different qubit systems, our architecture establishes a generic platform for exploring metropolitan-scale quantum networks.

## Teaser

Metropolitan-scale heralded entanglement between two independently operated quantum network nodes is achieved.

## INTRODUCTION

Future quantum networks distributing entanglement between distant quantum processors (*1*, *2*) hold the promise of enabling applications in communication, computing, sensing, and fundamental science (*3*–*6*). Over the past decades, a range of experiments on different qubit platforms have demonstrated the rudimentary capabilities of quantum networks at short distances including photon-mediated entanglement generation (*7*–*13*). These short-range qubit networks are useful for testing of improved hardware (*14*), developing a quantum network control stack (*15*), and for exploring quantum network protocols in a lab setting (*16*–*18*).

The next major challenge is to develop quantum network systems capable of generating, storing, and processing quantum information on metropolitan scales. Such systems face several previously unexplored requirements. First, the large physical distance, the consequential substantial communication times, and need for scalability demand that the network nodes operate fully independently. Second, as the optical fibers connecting nodes will extend for tens of kilometers, photon loss becomes a critical parameter that must be mitigated. Third, as advanced network applications require the heralded delivery of shared entangled states ready for further use, the qubit systems must be able to store quantum information for extended times, and the network system must be capable of applying real-time feedback to the qubits upon successful entanglement generation.

Recent qubit experiments have shown promising progress toward the latter two criteria, including the integration with efficient quantum frequency converters (QFCs) (*19*–*23*), demonstration of long coherence times on qubit systems that can be extended into multiqubit registers (*24*, *25*), and entanglement generation between nearby qubits via tens of kilometers of optical fiber (*26*, *27*). In parallel, experiments on ensemble-based quantum memories have pioneered notable advances on the first two criteria (*28*–*31*).

**Fig. 1.**
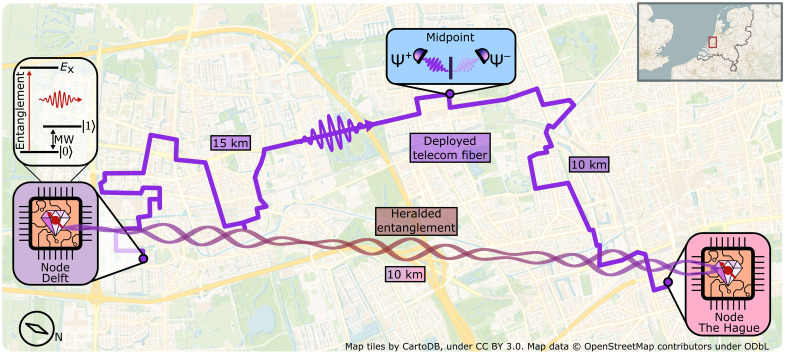
The metropolitan-scale quantum link. Cartographic layout of the distant quantum link and the route of the deployed fiber bundle, with similar quantum processor nodes in Delft and The Hague. Fiber length between node Delft and midpoint is 15 km and between node The Hague and midpoint is 10 km, with losses on the quantum channels at 5.6 and 5.2 dB, respectively. Inset to the quantum processor are the used qubit energy levels where the qubit is encoded in the electronic ground state addressable with microwave (MW) pulses, and the spin-selective optical transition (λ = 637nm) is used for entanglement generation and state readout.

Here, we report on the realization of a deployed quantum link between two solid-state qubit nodes separated by 10 km matching all three criteria. The two network nodes are combined with a midpoint heralding station via 25 km of deployed fiber, with all relevant classic and quantum signals propagating over the same fiber bundle in telecom bands (see Fig. 1). We implement an extensible architecture that enables the nodes to operate fully independently at large distance, mitigates the effects of photon loss on the entangling rate, and allows for full heralding of entanglement generation. Furthermore, the network architecture features precise polarization and timing control as well as active stabilization of the relative optical phase between photons emitted from the nodes, enabling the use of the loss-resilient single-click protocol for efficient entanglement generation (*32*, *33*). We benchmark the performance of the architecture by parameter monitoring and by generating entanglement in postselection. Last, we use the full network capabilities of heralding and real-time feedback to deliver entangled states shared between the nodes ready for further use. This demonstration establishes a critical capability for future applications and scaling and presents a key milestone toward large-scale quantum networking.

## RESULTS

### Deployed quantum network link architecture

**Fig. 2.**
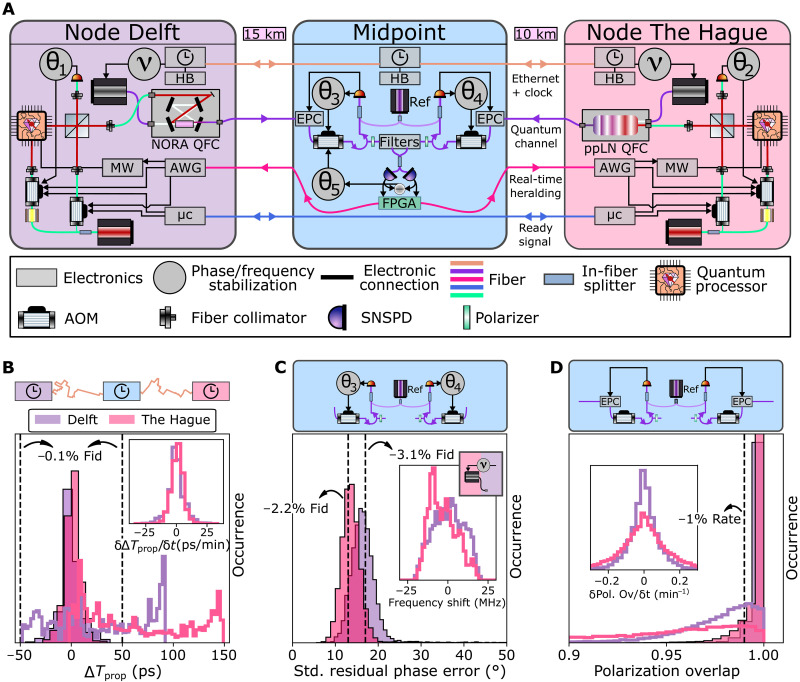
Quantum node components and metropolitan-scale stabilization performance. (**A**) Detailed components of the quantum nodes and fiber link connections. A microcontroller (μc) orchestrates the experiment, which, together with an arbitrary waveform generator (AWG), shapes laser- and microwave pulses, all synchronized by a heartbeat (HB) generator. Solid-state qubit entangled photon emission and stabilization light from each node is converted to the telecom L-band by the NORA (ppLN)–based QFC in node Delft (node The Hague) and sent to a central midpoint. There, long distance qubit-qubit entanglement is heralded via single photon measurement [superconducting nanowire single photon detector (SNSPD), efficiency ≈ 60%, darkcount rate ≈ 5 *s*^−1^] with detection outcomes fed back in real-time. The stabilization light is used for phase locking at the nodes (θ_1_, θ_2_), at the midpoint (θ_3_, θ_4_, θ_5_), phase-lock desaturation to the QFC pump lasers at the nodes (ν), and polarization stabilization via an electronic polarization controller (EPC). The performance of stabilization over the deployed link over 24 hours is shown for (**B**) time of arrival, (**C**) phase and frequency, and (**D**) polarization. Hardware providing active feedback (header) keeps these parameter that are drifting over time (line histogram) stable (shaded histograms) by enabling continuous feedback faster than the experienced drifts (insets). Vertical lines show the modeled impact on fidelity and rate.

To meet the challenges of metropolitan-scale entanglement generation, we designed and implemented the control architecture depicted in Fig. 2A. Each node contains a chemical vapor deposition grown diamond chip hosting a nitrogen-vacancy (NV) center electronic spin qubit that can be faithfully initialized and read out by resonant laser light and controlled using microwave pulses. The NV center optical transition at 637 nm is used for generating qubit-photon entanglement.

Each node is equipped with a stand-alone QFC unit that converts the 637-nm NV photons down to the telecom L-band at 1588 nm such that photon loss in the deployed fiber is minimized. The QFCs further serve as a tuning mechanism for compensating strain-induced offsets between the native emission frequencies typical for solid-state qubits. Through independent feedback on the frequency of the individual QFC pump lasers, we achieve conversion to a common target wavelength despite the few gigahertz difference in qubit emission frequencies (*34*). The QFC in Delft is based on a recently developed noise-reduced approach (NORA) (*35*) that produces two orders of magnitude lower background counts than the periodically poled Lithium Niobate (ppLN) with integrated waveguide-based QFC in The Hague (*36*).

To further mitigate photon loss, we use the single- click entangling protocol (*32*, *33*, *37*) which uses the number basis encoding for the photons. For this protocol, the entangling rate favorably scales with the square root of the photon transmission probability across the entire link, as opposed to schemes using photonic polarization or time-bin encoding which exhibit a linear scaling of rate with transmission (*7*–*10*, *12*–*14*, *26*, *27*). In the single-click protocol, each qubit is first prepared in an unbalanced superposition state $|\psi \rangle =\sqrt{\alpha }|0\rangle +\sqrt{1-\alpha }|1\rangle$. Application of an optical π-pulse resonant for qubit state ∣0〉 and subsequent spontaneous emission then results in qubit-photon entanglement, where the photonic qubit is encoded in the photon number state (0 or 1). Overlap of the photonic states at the beam splitter at the midpoint removes the which-path information, followed by single-photon detection by superconducting nanowire single-photon detectors. Upon measurement of one photon after interference, entanglement of the qubit states is heralded to $|\Psi^{\pm }\rangle =\left(|01\rangle \pm e^{\mathrm{i\theta}}|10\rangle \right)/\sqrt{2}$ with maximum fidelity 1−α, where the ± sign is set by which output arm the photon was detected in. The entangled state phase θ is dependent on the optical phase difference between the photonic modes arriving at the interference beam splitter from Delft and The Hague. Note that choosing the value of α involves a trade-off between higher signal to noise (larger α) and higher fidelity (smaller α). We use α = 0.25 in the current work.

This entanglement generation critically relies on photon indistinguishability at the heralding station, and therefore all degrees of freedom of the photons (frequency, arrival time, phase, and polarization) must be actively controlled at the metropolitan scale. A defining feature of our architecture is that stabilization laser light used for phase locking and polarization stabilization is time-multiplexed with the single-photon signals used for entanglement generation and sent over the same fiber from the nodes to the midpoint. To this end, we operate the link at a predefined heartbeat at 100 kHz, defining a common time division. During each heartbeat period of 10 μs, stabilization light is sent to the midpoint continuously, except for a 2-μs period where the entangling photonic states are sent out. This allows for near-continuous stabilization with high feedback bandwidth while performing entanglement generation. Below, we discuss the major sources of drift at metropolitan scale affecting the entangled state generation and our strategy for mitigating them.

First, length drift of the deployed fibers can result in reduced overlap of the photonic modes on the beam splitter. The timing of the optical π-pulse is locally controlled and disciplined by an optically linked distributed clock that doubles as Ethernet connection (White-Rabbit protocol) (*38*) over a dedicated fiber. Figure 2B shows a histogram of measured offsets in time of arrival for the two deployed fiber segments over 24 hours, with the inset displaying the drift speeds (σ = 5 ps min^−1^). By using these data to compensate for the drift via timing adjustments of the control electronics every ∼15 min, the offset is kept below 50 ps, much smaller than the photon 1/*e* decay time of 12 ns. The resulting entangled state infidelity due to length drifts is below 0.1%.

Second, as the phase difference of the photonic modes interfering at the beam splitter is imprinted on the entangled state, this phase must be known for the generated entangled states to be useful. To achieve a known and constant optical phase setpoint, five individual phase locking loops are implemented across the total link (see Fig. 2A). At each node, a local phase lock is closed between reflection of the resonant excitation light off the qubit device surface and the stabilization laser light via the controllers θ_1_ and θ_2_, stabilizing the in-fiber and free-space excitation and collection optics. Phase noise from the long fiber and excitation laser is mitigated by the controllers θ_3_ and θ_4_ via interference of light from the midpoint reference telecom laser and frequency down-converted stabilization light at nanowatt levels. Analog phase feedback is performed locally at the midpoint directly on the incoming light yielding a high stabilization bandwidth exceeding 200 kHz. Last, interference of telecom stabilization light from both nodes at the central beam splitter is measured by the single-photon detectors and input to controller θ_5_, closing the global phase lock between the nodes (see the Supplementary Materials for additional details). We note that the modularity of this architecture directly allows for the connection of multiple nodes to the same midpoint in a star topology, as the synchronization of all incoming signals to a central reference and relative phase stabilization between links can be performed using the control system at the midpoint.

Figure 2C displays a histogram of the resulting phase errors during 24 hours of operation. Stable operation is achieved with a few-percent impact on the entangled state fidelity per connection. As this architecture yields full control over the phase difference at the beam splitter, the phase θ of the entangled state can be tuned on demand by adjusting the setpoint of the phase lock (see the Supplementary Materials). To maintain the phase lock under frequency drifts at the nodes, two individual feedback loops (ν) between the nodes and the midpoint adjust the frequency of the individual QFC pump lasers at an update rate of 500 Hz. This desaturation allows for a large dynamic range of the phase feedback, as required to handle the observed frequency drift range of >50 MHz (see Fig. 2C, inset).

Third, polarization drifts, although considerably slower than phase drift, must also be mitigated. To this end, the stabilization light is additionally used for electronic polarization compensation at the midpoint. The amplitude of the error signal input at θ_3_ and θ_4_ is dependent on polarization overlap with an in-fiber polarizer. We use this as input for a gradient ascent algorithm to feedback on the polarization of the incoming light at the midpoint. Data on the deployed link (Fig. 2D, inset) show that polarization drift occurs on second timescales; our feedback at a few hertz bandwidth keeps the polarization aligned to within a few percent (Fig. 2D). Any remaining polarization mismatch is removed by the polarizers at the cost of a slightly reduced entanglement generation rate.

### Postselected entanglement generation over a deployed link

We now turn to the performance of the deployed link in generating entanglement between the solid-state qubits at the remote nodes. The proper functioning of all components of our system is first validated in a set of experiments with all devices of the link in a single lab in Delft, showing successful entanglement generation at state fidelities exceeding 0.6 (see fig. S3). After connecting and calibrating the equipment at the remote locations, we first focus on generating entanglement in postselection. In this protocol, the qubits are measured directly after generating spin-photon entanglement, and successful photon detection at the midpoint is used in postprocessing to analyze entanglement generation. This scenario is compatible with quantum key distribution but does not allow for more advanced protocols as the entangled state is not available for further use (*2*).

**Fig. 3.**
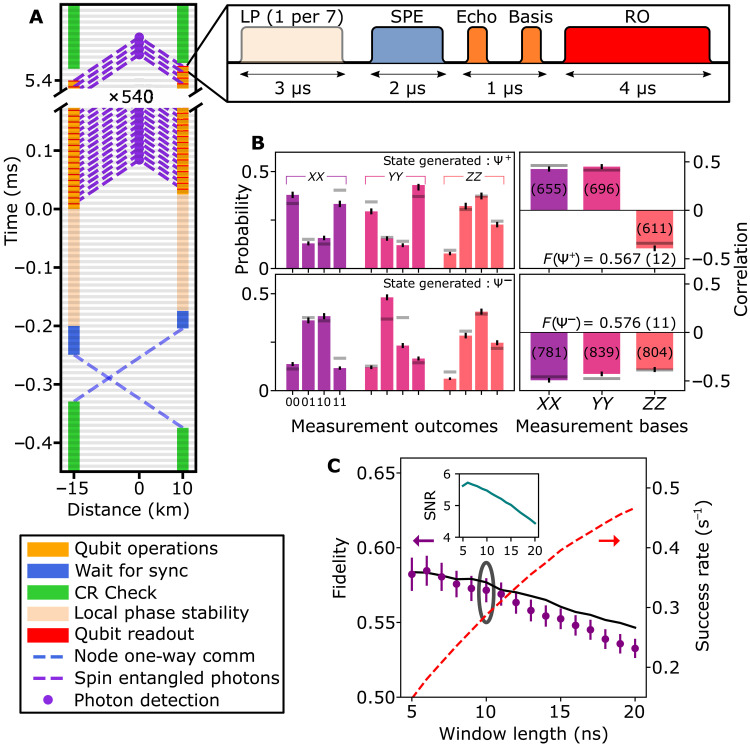
Postselected entanglement over the deployed link. (**A**) Space-time diagram depicting the generation of entanglement in postselection. Horizontal gray lines indicate the periodic heartbeat of 100 kHz. Local qubit control used to generate entanglement and perform state readout (pop-out) all fit within one heartbeat period of 10 μs. A local phase (LP) pulse is followed by spin-photon entanglement (SPE) generation, an echo, and basis selection microwave pulse. Last, the state is readout (RO). (**B**) Outcome of correlation measurements, with different detector signature for the top and bottom panels. We show the qubit-qubit readout outcomes per correlators (left), as well as the resulting values per correlator (right). The calculated state fidelity is given inside each figure. The number in parentheses indicates the amount of events recorded for that correlator. Horizontal gray bars indicate the theoretical model. (**C**) Average state fidelity (left vertical axis) and entanglement generation rate (right vertical axis) for varying photon acceptance window length. Circling indicates the window used in (B), and the black solid line is a model (see the Supplementary Materials). Inset shows signal-to-noise ratio for the various window lengths. All measurement outcomes are corrected for tomography readout errors, and error bars are 1 SD.

Our implementation of the postselected entanglement generation is depicted in the space-time diagram of Fig. 3A, where each horizontal gray line depicts one 10-μs heartbeat period. Both nodes signal their start of experiment after passing their own charge-resonance check (CR Check) that ensures that the lasers are on resonance with the relevant optical transitions (*16*). After communicating their readiness, they resolve the earliest heartbeat to start attempting entanglement generation. In the first 20 heartbeat periods, both nodes stabilize their local phase, followed by 540 rounds of entanglement generation, with one attempt per heartbeat period. Every seventh entanglement round also contains optical pulses for maintaining stability of the local phase. The operations performed at the nodes for each round are detailed in the pop-out of Fig. 3A. The sequence returns to the CR Check after completing the preset number of rounds.

We characterize the generated nonlocal states by measuring qubit-qubit correlations in different readout bases. In Fig. 3B, we plot the outcomes for the three basis settings split out per detector, showing the expected (anti-)correlations. Combining the outcome probabilities, we calculate the correlators 〈*ZZ*〉, 〈*XX*〉, and 〈*YY*〉. Note that as the two detectors herald different Bell states (Ψ^+^ versus Ψ^−^), the corresponding 〈*XX*〉 and 〈*YY*〉 correlations have opposite sign. We also plot the values predicted by our detailed model without any free parameters (gray lines, see the Supplementary Materials) and observe good agreement with the data. The asymmetry in the amount of events is caused by a difference in quantum efficiency between the two single-photon detectors. We find that the measured fidelities $F\left(|\Psi^{\pm }\rangle \right)=\frac{1}{4}*\left(1-\langle \mathrm{ZZ}\rangle \pm \langle \mathrm{XX}\rangle \pm \langle \mathrm{YY}\rangle \right)$ with respect to the ideal Bell states are significantly above 0.5, proving the generation of postselected two-qubit entanglement (Fig. 3B).

In the above analysis, we included photon emission up to 10 ns after the optical π-pulse. By varying the analysis window of the photon detection, we can explore the trade-off between rate and fidelity (Fig. 3C). We find that the entangled state fidelity slowly decreases with increasing window length, due to decreasing the signal-to-noise ratio of the photon detection at the midpoint (see the inset in Fig. 3C) in addition to the window size–dependent influence of spectral diffusion (*34*, *37*). Other examples of sources of infidelity are the residual optical phase noise and the probability of double optical excitation, all taken into account by our model (black line, see the Supplementary Materials). At the same time, as more photon detection events are accepted with increasing window length, the success rate increases. The achieved entanglement generation rate reaches 0.48 Hz (success probability per attempt of 7.2 ⋅ 10^−6^) for a 20-ns window.

### Fully heralded entanglement generation over a deployed quantum link

In a final demonstration that highlights the capabilities of the deployed platform, we generate fully heralded qubit-qubit entanglement. In contrast to the postselected entanglement generation described above, “live” entangled states are now delivered to the nodes that can be further used for quantum information tasks. Such live entanglement delivery is a fundamental requirement for many future applications of long-range entangled states (*2*).

**Fig. 4.**
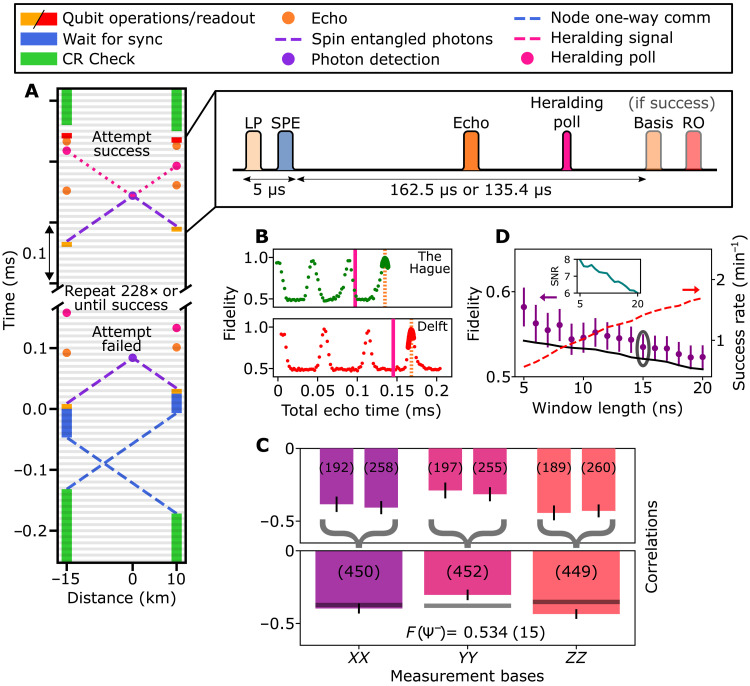
Fully heralded entanglement over the deployed link. (**A**) Space-time diagram of fully heralded entanglement generation. An attempt is successful upon registering a heralding signal at the polling time, after which a feed-forward is applied on the qubit, and readout is performed. The absence of a heralding signal communicates a failed attempt, where we retry for a maximum of 228 attempts or until success. Pop-out depicts the local qubit control, basis selection, and readout pulses. The time between spin-photon entanglement, heralding poll and basis selection is node dependent. (**B**) Hahn-echo experiment on the communication qubit, showing the revivals of the coherence (*39*). The solid vertical line indicates the heralding poll, and the dotted line indicates the time of the basis selection. All times are with respect to echo sequence start. (**C**) Correlation measurement for full heralding, showing both detector outcomes delivering the same Ψ^−^ state. Top (bottom) plot shows events per detector (combined). Bars indicate data, and the number in parentheses indicate the amount of events. Horizontal lines indicate the theoretical model. (**D**) Average state fidelity (left axis) and entanglement generation rate (right axis) for varying photon acceptance window length. Circling indicates the window used in (C), and the black line is a model (see the Supplementary Materials). Inset shows signal-to-noise ratio (SNR) for respective window lengths. Measurement outcomes are corrected for tomography readout errors, and error bars are 1 SD.

We emphasize that this protocol requires that all relevant heralding signals (including which detector clicked) are processed at the node before the entanglement delivery is completed. To this end, we use the experimental sequence depicted in Fig. 4A. To preserve the qubit states with high fidelity while waiting for the heralding signals to return and be processed, the refocusing echo pulse is applied to the qubits halfway the sequence to dynamically decouple them from spin bath noise in their solid-state environment. Figure 4B shows the resulting qubit preservation as a function of time depicting periodic revivals of coherence due to interactions with nearby nuclear spins (*39*). We note that with established advanced pulse sequences, these revivals can be set with high timing resolution, and the NV qubit coherence time can be extended toward a second (*40*). While the qubits are protected at the nodes, the photons travel to the midpoint in about 52 and 73 μs from The Hague and Delft, respectively. A field-programmable gate array (FPGA) at the midpoint processes the output of the single-photon detectors, establishing whether a photon was detected in a predetermined time window and in which detector. The electronic output of the FPGA is optically communicated to the nodes, taking another 52 μs (73 μs). There, the signal is detected and processed live to choose the next action. The time at which this final processing is completed per node is indicated by the pink solid lines in Fig. 4B.

We choose to use the first echo revival (orange dotted vertical line in Fig. 4B) after the expected arrival time of the heralding signal to complete the delivery of the entangled state. For these setpoints, a detailed measurement of the echo contrast shows that the wait times introduce a 1.3 (3.2) percent reduction in coherence for node Delft (The Hague).

The entanglement generation runs are automatically repeated by the nodes until a successful heralding signal is received from the midpoint. Once such a signal is received, the system jumps to a different control sequence in which a basis selection gate is applied to each qubit followed by single-shot qubit readout. This control sequence incorporates the which-detector information communicated from the midpoint in real time. We exploit this full heralding capability to apply a phase flip conditioned on which detector clicked, thereby delivering the same Bell state $\Psi^{-}=1/\sqrt{2}\left(|01\rangle -|10\rangle \right)$ for each of the two possible heralding signals.

The measured correlations per readout basis are shown in Fig. 4C, showing the expected anticorrelated outcomes for all three bases. The top bars show the outcomes divided per detector, displaying now the same Ψ^−^ thus showing the successful operation of the real-time feedback. We find that the delivered entangled states have a fidelity of 0.534 (*15*). This result establishes the first demonstration of heralded qubit-qubit entanglement at metropolitan scale, with all the heralding signals processed in real-time and the entangled states delivered ready for further use.

Figure 4D displays the rate-fidelity trade-off in analogy to Fig. 3C, showing a similar trend. The reduction in rate (0.022 s^−1^ = 1.3 min^−1^ at the predefined 15-ns window length) compared to the postselected case is mainly due to the added communication delay needed for the heralding signal to travel to the nodes, making each attempt a factor of ≈20 slower. The observed fidelity versus window size is well captured by our models, with the reduction in fidelity compared to the postselected case mainly coming from the additional decoherence and a reduced phase stability (black line, see the Supplementary Materials). The improved signal-to-noise ratio (Fig. 4D, inset) compared to Fig. 3C is due to an improved trade-off between detection efficiency and dark counts following an optimization of the single-photon detector bias currents.

## DISCUSSION

We have realized a deployed quantum link and demonstrated heralded entanglement delivery between solid-state qubits separated at metropolitan scale. The architecture and methods presented here are directly applicable to other qubit platforms (*13*, *26*, *41*–*44*) that can use photon interference to generate remote entanglement and frequency conversion to minimize photon losses. In addition, the ability to phase-lock remote signals without the need for ultrastable reference cavities can be of use for ensemble-based quantum memories (*28*–*30*, *45*).

This work can benefit from future developments in the following ways. Real-time correction of false heralds (*17*) can be realized by using detection events of phonon side-band photons on the nodes, upon which the fidelity of the delivered entangled state improves (see the Supplementary Materials). Near-term developments can substantially improve the signal-to-noise ratio, which is now limiting the entangled state fidelity (about 30% contribution) through false heralding events and forcing a high value of α (protocol error). For instance, the signal can be substantially boosted by embedding the NV center in an open microcavity (*46*, *47*) or by using different color centers that exhibit a more efficient spin-photon interface (*23*, *27*, *48*, *49*). As a quantitative example, replacing the NV center with a diamond Tin Vacancy (SnV) center (which has a 16 times higher probability of coherent photon emission), using NORA QFCs at all nodes (*35*) (here used only in the Delft node), and fixing a known imperfection in the local phase stabilization would reduce the residual phase noise, already increasing the heralded state fidelity to above 80%. Furthermore, improving coherence protection using XY8 sequences and systematic reduction of the remaining small error sources could lift the fidelity beyond 90% (see table S4). The extensible nature of our architecture opens the door to connecting more than two qubit nodes to a midpoint without additional overhead by locking to the same reference, which, in combination with using local memory qubits (*14*, *24*, *50*, *51*), would enable the exploration of more advanced protocols on a metropolitan-scale network (*17*, *18*, *52*), as well testing quantum control stacks (*15*) on a distributed multinode quantum network.

## MATERIALS AND METHODS

The experimental setups used in this work are built on top of the hardware described in (*34*). A detailed schematic of the quantum nodes, midpoint, and their connections is shown in Fig. 2. Hardware control is enabled through use of software based on the Quantum Measurement Infrastructure, a Python 3 framework for controlling laboratory equipment (*53*). We will give a brief overview of the relevant parts of the setup below.

### Quantum node

Each node houses a diamond-based quantum processor consisting of a NV center electronic spin qubit in the negative charge state. The ground-state spin levels are split using a small permanent magnetic field aligned with the NV axis of ≈3 mT, allowing for arbitrary qubit rotations with a microwave pulse frequency of ≈2.8 GHz. Initialization, readout, and qubit-photon entanglement generation is achieved through resonant excitation at 637 nm.

### Quantum frequency converters

We use both an in-house built ppLN QFC module and the NORA QFC described in (*35*). The NORA QFC mitigates the amount of noise photons generated by the frequency conversion due to imperfections in the waveguide and poling period of ppLN crystals. We compare the NORA QFC to the ppLN QFC in table S1 and refer to (*35*) for more information.

### Phase stability

To allow for phase stability between physically separated setups, we use a prototype TOPTICA DLC DL pro 637 nm using optical feedback from an additional cavity to reduce the phase noise to <40 mrad integrated from 100 kHz to 100 MHz. We use a combination of five interferometers with heterodyne detection and feedback that together lock the phase to a controlled setpoint. On both nodes, we define local interferometers that lock the excitation laser path to the stabilization path of the respective setups. On the midpoint, we stabilize the incoming light from each node to the same telecom wavelength reference. The relative optical phase between the nodes is stabilized using interference at the central beam splitter in the midpoint and measured with superconducting nanowire single photon detectors (SNSPDs). The optical fields and respective frequencies used are shown in fig. S1, and the combination of the stabilization fields with the NV control fields is shown in figs. S4 and S5. More detail on the phase stability implementation can be found in sections S1.1 to S1.5 and S2. In addition to phase stability, the midpoint provides polarization control, spectral filtering, and single-photon detection (section S4).

### Midpoint

The midpoint provides phase feedback, polarization control, spectral filtering, and single-photon detection. The phase feedback uses frequency modulation of acousto-optic modulators for phase locking of the node excitation lasers to the midpoint telecom reference at ≈1588. Two electronic polarization controllers allow for full control over the polarization state to compensate for fiber drifts. Two low-loss variable optical attenuators shield the SNSPDs from bright stabilization light coming from the nodes. Per node, all the error signals for the phase and polarization stabilization and temperature of the ultranarrow fiber Bragg-grating filters are generated on the same balanced photodiode and subsequently extracted by a combination of power splitters, bandpass filters, and amplifiers. An FPGA development board processes electrical signals to allow for real-time heralding to the nodes of photon detections at the midpoint.

## Supplementary Materials

### This PDF file includes:

Supplementary Text Figs. S1 to S11 Tables S1 to S4 References
