# Supplementary material for: Metropolitan-scale heralded entanglement of solid-state qubits
Source: Sci Adv. 2024 Oct 30;10(44):eadp6442. doi: 10.1126/sciadv.adp6442 (PMC11524177; doi:10.1126/sciadv.adp6442)
Supplement: Supplementary file 1 — Supplementary Text Figs. S1 to S11 Tables S1 to S4 References [file sciadv.adp6442_sm.pdf]

Science Advances
AAAS

Supplementary Materials for

Metropolitan-scale heralded entanglement of solid-state qubits

Arian J. Stolk *et al.*

Corresponding author: Ronald Hanson, r.hanson@tudelft.nl

Sci. Adv. **10**, eadp6442 (2024)
DOI: 10.1126/sciadv.adp6442

This PDF file includes:

Supplementary Text
Figs. S1 to S11
Tables S1 to S4
References

S1 Experimental setup

The experimental set-ups used in this work are built on top of the hardware described in Ref. (*34*) and they use software based on the Quantum Measurement Infrastructure, a Python 3 framework for controlling laboratory equipment (*53*). In this section we will discuss the hardware changes made that enabled compatibility with metropolitan-scale entanglement gen-eration.

S1.1 Quantum Node

We exchanged the diamond quantum device of Node The Hague for a newly fabricated one. All parameters of the substrate (Element Six) and device, such as purity, crystal orientation and carbon isotopes are the same as previously reported. To allow for phase stability between physically separated setups, the TOPTICA DLC DL pro $637\,\mathrm{nm}$ was exchanged for an upgraded prototype employing optical feedback from an additional cavity to reduce the phase noise to $< 40\,\mathrm{mrad}$ integrated from $100\,\mathrm{kHz}$ to $100\,\mathrm{MHz}$.

S1.2 Quantum Frequency Converters

As mentioned in the main text, we have replaced one of the in-house built QFC modules with the NORA QFC described in (*35*). This design mitigates the amount of noise photons generated by the frequency conversion due to imperfections in the waveguide and poling period of periodically poled Lithium Niobate (ppLN) crystals. Briefly, the novel approach consists of identifying the right bulk material that can be critically phase-matched to perform efficient conversion, and embedding the crystal in a single-resonant cavity to enhance the effective pump power in the conversion process. We compare the NORA QFC to the ppLN QFC in Table S1., and refer to (*35*) for more information.

S1.3 Phase stability on the nodes

On both nodes we added the components to define the local interferometers that lock the excitation laser path to the stabilization path of the respective set-ups. We use a combination of up- and down-shift Acousto-Optical Modulators (AOM, Gooch and Housego Fiber-Q), already used for amplitude modulation, to define a $400\,\mathrm{MHz}$ frequency offset. The excitation light reflected off the diamond surface is separated from the zero-phonon-line (ZPL) path by a polarizing beam splitter (PBS), where it spatially overlaps with the stabilization light. We guide the light to a set of birefringent crystals that maximize the complex overlap between the orthogonally polarized reflected beam and the stabilization light. A polarizer then selects a common polarization such that both beams can interfere in intensity, after which we focus the light into a multi-mode fiber (core diameter $\approx 100um$) and measure the interference beat signal using a fast avalanche photodiode (Menlo Systems APD210). After amplification and filtering, we extract the phase of the interference by mixing it with an electronic reference signal generated by a stable RF source (Anapico APSIN6010). The feedback signal generated is passed through a Track-And-Hold amplifier (Texas Instruments OPA1S2384) and input to the Frequency Modulation input of the Excitation light AOM RF-driver (TimeBase DIM3000), which closes the feedback loop. Because the frequency modulation has integral feedback on the phase that can not be switched on/off fast, any bias/offset in the error-signal reduces the free evolution time during which the local phase remains stable. In a future implementation, this actuator will be replaced by a linear phase shifter, and an improvement of the local phase-stability can be realized.

S1.4 Midpoint

The previous design of the midpoint used for two-photon quantum interference (*34*) suffered from an increased noise floor due to the presence of bright laser pulses incident on the nanowires every $200\,\mu\mathrm{s}$. To reach an adequate feedback bandwidth for phase-locking of our excitation

lasers to the telecom reference, we have to reduce the period of bright pulses to $10\,\mu s$, making this even more challenging. We have made the following improvements to allow for polarization and fast phase feedback, to reduce crosstalk between optical channels and lower the background counts in the SNSPDs:

- Split the high-power reference light into three paths, two used to generate an optical beat to lock the stabilization light coming from each node to the reference laser, and one used to generate an error signal to stabilize the ultra-narrow Fibre-Bragg grating filters.
- Added two low-loss Variable Optical Attenuators (VOAs, Boston Applied Technologies Nanona VOA) that shield the detectors from bright pulses coming from the nodes.
- Added two Electronic Polarization Controller (OZOptics EPC-400-11-1300/1550-9/125-S-3A3A-1-1) to allow for full control over the polarization state to compensate for fiber drifts.
- Added two AOMs to have fast and integral control of the phase of the incoming light, used for phase stabilization.
- Removed one circulator from the design and splicing multiple connectors where the single-photons pass through to reduce single-photon loss

All the error signals for the phase- and polarization stabilization, and the ultra-narrow fiber Bragg-grating (FBG) for one node are generated on the same balanced photodiode (Thorlabs PDB480C-AC), and subsequently extracted by a combination of power-splitter, bandpass filters and amplifiers.

S1.5 Heralding

To allow for low-latency feedback of photon detections at the midpoint, the midpoint was upgraded with an FPGA development board (Digilent Arty-A7) with custom-built PCBs converting the external pin I/O to SMA connectors with the possibility of 50 Ohm matching. Single photon signals were input on a custom pulse-stretcher, cleaning up the electrical pulse from the SNSPD to a block-like pulse shape of around $1\,\mu s$ and input on the I/O of the FPGA. The complete I/O of the FPGA is shown in Fig. S9. The acceptance window of sending out a heralding signal is predetermined at the time within a heartbeat period where we expect spin-photon entangled photons to arrive. The exact timing of the acceptance window is derived from the centralized clock and heartbeat signal available at the midpoint. To be able to signal which detector clicked on a photon count, we use two digital pulses to herald, one for the success, and one for which detector clicked. Since the delay in time-of-flight between the nodes is different, the digital pulses need to be aligned individually to arrive at the nodes at the same time within one heartbeat. To clarify, this does not mean they arrived at the same global time at the nodes, but that the digital pulses arrive at the same time modulo the heartbeat ($10\,\mu s$) at the nodes. Lastly, a local FPGA code loading server was employed that could upload compiled-VHDL that was either manually or automatically generated remotely. A change in timing of sending out pulses could be updated on the underlying VHDL code automatically based on a custom template code. This allowed fast multi-core compilation locally and remote upload to considerably reduce debugging time when determining the required exact timing of the heralding pulses.

S2 Phase stabilization in the midpoint

We stabilize the incoming light from each node to the same telecom reference (fast stabilization). The relative optical phase between the nodes is stabilized using interference at the central beam splitter in the midpoint, and measured with the SNSPDs (global stabilization). The optical fields and respective frequencies are shown in Fig. S1, and the distribution of the fields in time shown in Fig. S4 and S5.

S2.1 Fast stabilization

Part of the stabilization light used in the local phase lock also propagates via the QFC, through the same deployed fiber, to the midpoint. There it is split off in frequency from the single-photons via the FBG, subsequently interfered with a telecom reference laser (NKT Adjustik fiber laser), where a heterodyne interference beat is measured by the balanced photodiode. A phase-frequency detector (Analog Devices HMC3716LP4E) processes this signal by comparing it to a reference signal (Wieserlabs FlexDDS-NG DUAL). Its output serves as the error-signal for a high-speed servo controller (Newport LB1005-S) performing PID control. This controller provides feedback on the AOM to stabilize the incoming light, synchronizing it with the reference at a bandwidth of over $200\,\text{kHz}$. To stay within the low-loss performance of the AOM close to the central frequency, we offload the accumulation of phase error (frequency drifts) to the QFCs at the nodes. This can be done much slower ($500\,\text{Hz}$) and makes the feedback only limited by the frequency range of the QFCs ($\gg 1\,\text{GHz}$). The fast feedback removes all high-frequency noise from the incoming light, which contains the excitation laser line-width, as well as phase noise introduced by expansion/contraction and vibrations of the deployed fiber.

S2.2 Global stabilization

To further compensate phase drift that occurs in the midpoint and to set the relative phase between the incoming optical modes, we employ a control loop based on the stabilization light from both nodes that interferes at the central beam splitter. This light leaks through the FBG, and is of sufficient low power to not blind the SNSPDs. The voltage pulses from the SNSPDs are amplified, and a difference amplifier (Krohn-Hite Model 7000) generates a heterodyne beat by subtracting the count-rates from each detector. This beat is compared to a reference signal (AimTTi) by mixing it and low-pass filtering (150 Hz), generating an error-signal. This is again input to a servo controller (Newport LB1005-S), of which the output is used to modulate the phase of the reference of one of the fast stabilization controllers.

S2.3 Verifying phase stability

We can verify the performance of the full optical phase stabilization by interfering the two excitation lasers from the nodes at the central beam splitter in the midpoint, using the time-resolved counts in the SNSPDs to generate the error signal. By changing the phase of the reference signal, we can measure a full visibility fringe of the interference. By correcting the measured interference for the imbalance in photon flux from each node, we can calculate the interference contrast, see Fig. S2B. A more in-depth description and technical analysis of the total phase stabilization will be given in a separate manuscript (*54*).

S3 Overview communication times and decisions

In this section we discuss the different optical and electrical pulses or feedback signals that are needed to control this distributed experiment. We will first discuss the local nodes, then which signals are necessary to be timed at the midpoint for single photon processing and phase stability. Lastly, we will discuss how we align the arrival time of the photonic states at the

midpoint telecom detectors.

S3.1 Pulses and timing at node

Since the central clock in the midpoint is distributed to the nodes by the White Rabbit enabled switches, it provides a common reference frame to align the time-of-arrival for each of the entanglement generation attempts, see Section S3.3.

A full timing overview of a single heartbeat length when we perform a full entanglement attempt without heralding is shown in Fig. S4A and with heralding in Fig. S4B. The entanglement generation attempt with heralding contains exactly the same pulses as in Fig. S4A, except for that the time between the spin superposition state generation and basis-selection pulse is at a revival of the spin coherence, as previously shown in the pop-out of Fig. 4A of the main text.

S3.2 Pulses and timing at midpoint

The laser, electrical and feedback pulses used at the midpoint by design operate agnostically of the experiment that is being run. For about $\sim$8 µs we can perform phase, frequency and FBG stability actions using the interference from the reference light and the incoming stabilization light from the node (see Fig. S5). In the remaining $\sim$2 µs the local reference light is shut off, the phase Track-and-Hold amplifier is set to 'hold' and the VOAs opened fully to allow for single photons to pass through to the SNSPDs.

S3.3 Time-of-arrival alignment

One of the requirements for indistinguishability of the photons is the time aligned photonic mode overlap at the beam splitter in the midpoint. The timing of the emission of the photonic state determines, together with the time of flight (ToF), the time at which the photonic state arrives from the node at the midpoint. This is especially crucial in the metropolitan distance case, where fiber distance varies with tens of picoseconds per hour (see Table S2). Since we have

unbalanced fiber connection lengths between the nodes-midpoint, Node Delft will be allowed to send its photon as soon as possible. Node The Hague has to hold its pulse sequence start for 3 heartbeats. Both nodes require sub-heartbeat timing alignment to align the arrival time of the solid-state entangled photons in the last $\sim 2\,\mu s$ of the midpoint scheme (Fig. 5). This last alignment step is achieved with the arbitrary waveform generator (AWG, Zurich Instruments HDAWG) sequencer and the built in skewing parameter of the AWG outputs.

Due to the constantly changing ToF, the alignment in time is performed in two steps. A first time alignment is required to be performed manually where laser pulse timing is recorded at the midpoint timetagger (PicoQuant Multiharp, $80\,\mathrm{ps}$ resolution), after which the time difference between arrival times of the pulses from the two nodes can be calculated and adjusted. This value is recorded as calibration parameter. Secondly, the reported ToF from the time synchronization system is used to automatically adjust the skewing parameter every $\sim$15 minutes with the difference in fiber ToF with respect to the initial calibration.

S4 Other midpoint stabilization

S4.1 Polarization stabilization

We continuously stabilize the polarization of the incoming stabilization light using the same beat generated on the balanced photodiode as mentioned in the phase stability section (Section S2) . Because of our in-fiber polarizer, the amplitude of the beat will depend on the overlap of the incoming light with the polarizer. Maximizing this amplitude will guarantee the same polarization between the nodes, as needed for interference. Using a phase-gain detector (Analog Devices EVAL-AD8302) we can monitor the amplitude of the beat by comparing it to an in-phase RF tone (generated by Wieserlabs FlexDDS-NG DUAL) over multiple orders of magnitude. We sample the output of this phase-gain detector with an Analog Discovery 2 (Digilent), and process the error signal in a python process running a Gradient Ascent algorithm. This in

turn sends commands to the EPC that adapts the polarization state and completes the control loop. The whole process can be monitored and adjusted via a Graphical User Interface.

S4.2 Ultra-narrow FBG stabilization

We adapted the control loop for the temperature stabilization of the ultra-narrow FBGs (UNF) to allow for a considerable reduction of the optical power reflected back to the S NSPDs. We achieved this by using a heterodyne beat measurement that is sensitive to the transmission through the UNF, instead of a simple power measurement at DC. This allows for multiple orders higher sensitivity, and allows us to stabilize the filters with only $\approx 10\,\mathrm{fW}$ of light traveling through the filter in the backwards direction.

S5 Deployed fiber characterization and stability

We use a fiber bundle with four fibers to perform all the communication between the locations. These are provided to us by KPN, a major Dutch telecom provider, and are part of their telecom infrastructure. The reality of using deployed fibers means that it is built up of sections of continuous fiber, which are spliced or connected together at various locations along the path. Therefore the losses per kilometer of deployed fiber are notably higher than the ideal losses of $\approx 0.2\,\mathrm{dB\,km^{-1}}$ @ $1580\,\mathrm{nm}$, and can differ from fiber to fiber. During the period of measuring over the deployed link in 2023, several parts of the fiber were adapted and re-routed as part of restructuring of the network by KPN, changing both their length ($\pm$ $100\,\mathrm{m}$) and loss ($\pm$ $1\,\mathrm{dB}$) multiple times over the span of a few weeks. Table S2 shows typical loss values measured during this period. Thanks to the modular approach of our system, we have complete freedom in changing the arrival time of the photon in the midpoint, allowing for easy re-alignment of the propagation delay, both for the fiber restructuring as well as the daily expansion and contraction.

S6 Calibrations

S6.1 Entanglement generation

We employ a method where we interleave the generation of entanglement with three calibrations that measure and re-calibrate critical parameters and settings. These calibrations are an adapted version of the entanglement generation sequence that measure specific key elements of the entanglement generation: the signal-to-noise ratio (SNR), optical phase stability (PHASE) and the entangled state phase (XsweepX). Typical results of these three calibrations are shown in Fig. S2. When these calibrations have passed their threshold, we proceed to measure the correlators used to calculate the entangled state fidelity, FID for short.

The first c alibration i s t he m easurement o f t he s ingle-photon s ignal f rom b oth n odes and the background, which includes all the losses that are present in the system. We prepare the communication qubit in the $|0\rangle$ to maximize the single photon signal. The measurement is the same experimental sequence as shown in Fig. S4A, where now the state generation α pulse is removed to measure the signal, and replaced by a π-rotation to measure the noise. Furthermore, the arrival time of the photons is shifted by $100\,\mathrm{ns}$ to measure the brightness of both nodes individually.

Second is determining the contrast and phase of the relative optical phase between the nodes. We realize this by interfering a bright pulse (1 μs) of the excitation lasers which we reflect off the diamond surface, and follows the exact path as the NV-center emission from there. By varying the setpoint of the phase in the stabilization scheme, we can measure a full interference fringe at the SNSPDs as seen in Fig. S2B and characterize the residual phase noise of the system at the specific time window where single photons interfere.

Furthermore, this calibration is used to set the phase of the optical interference at any point along this fringe, which can be used to optimize the entangled state fidelity (*37*). We show the

capability of changing the optical phase to two different setpoints of 0° and 180°.

The third and last calibration is the measurement of the entangled state phase, which has an offset with respect to the optical phase setpoint. Using the single-click protocol we create a correlated spin-spin state that we can use to probe this phase. We do this by measuring correlations in the rotated basis X on Node Delft, while varying the readout basis in the Bloch sphere equator plane for Node The Hague. We can extract this entangled state phase by fitting the resulting oscillation with a single cosine, see Fig. 2C. We feed-forward this phase in the entanglement measurements to always generate the $\Psi^{\pm} = \frac{1}{\sqrt{2}}\left(|01\rangle \pm |10\rangle\right)$ during the entanglement generation.

S6.2 Experimental setup performance

We maintain similar performance parameters of the experimental setup by performing calibrations of many different settings at different timescales. On a daily timescale, performance parameters of the communication qubit are re-calibrated:

1. Laser power (see Fig. S10)
2. Single-Shot Readout
3. Microwave qubit control (π, $\pi/2$, α)
4. Optical excitation (optical π pulse) for entanglement generation attempt
5. QFC efficiency

Roughly every 20 minutes, a set of shorter timescale calibrations are run. Some are dependent on performance parameters that are available live during or right after a measurement set. See Fig. S10 for the flowchart showing the calibrations and decisions made. As discussed in the previous section, we require several types of measurements to calibrate the phase of the

entangled state. If in any of those measurements we are outside of the bound of acceptance, we restart the sequence. Additionally, the FID measurement sequence is cut into smaller blocks of 10k (30k) successful CR-checks for the heralded (delayed-choice) metropolitan distance experiments. After each block, the CR-check passing rate is checked against the average CR-check rate during the SNR sequence (per node). If the passing rate is below this value, we exit the FID sequence and continue. We do this because the CR-check passing rate is a value that includes many derived underlying performance parameters of the setup as a whole, e.g. laser wavelengths, laser power, objective position and others. If any of those values changed substantially the CR-check passing rate is negatively impacted, which we use as a signal that an underlying parameter of the experiment is drifting off ideal and subsequently we break out of FID data taking. As can be seen in the flowchart, afterwards all calibrations are restarted, and we start from a calibrated setup with the SNR sequence, which is when we expect the highest CR check passing rate, and as such, use that to define our threshold for the rest of the experiment.

S7 Modeling the generated entangled state

To assist in setting up the experiment and simulating the outcomes, we employ a Monte-Carlo simulation based on the model described in (*37*). This is an extensive description of generating entanglement between remote nodes using the single-click protocol. It considers the spin-spin density matrix, based on the detection events where at most two photons reach the central detectors. By calculating the probability of each detection pattern and its corresponding spin-state, it arrives at the average density matrix given non photon-number resolving detectors. It takes into account many physical parameters that have an impact on the entangled state fidelity, such as photon loss, photon indistinguishably, and the photon wavepacket shape. It also includes several experimental parameters such as the effects of residual phase noise, dephasing noise and darkcount probability. We noticed that in contrast to the $13\ \mathrm{MHz}$ Full-Width Half Maxi-mum (FWHM) spectral diffusion found in (*37*), a FWHM of $25\ \mathrm{MHz}$ represents the fidelity vs. window length trend better.

In the experiments presented in the main text we do not apply a post-processing correction using the Charge-Resonance check after the readout of the spin-state. This correction can be done to identify sequences where one of the nodes is in the NV^0 state, and no longer optically responsive to the excitation laser, but only in a post-processed fashion. To accurately model the effect of including events when the NV-center is ionized on the fidelity with the ideal state, we extend the model by including the probability of one of the nodes being ionized. We assume that, when an NV photon from a particular node in the midpoint is detected, this node can not be in the ionized state, but the other node can be with a probability p_{ion}. If the event in the midpoint is due to a noise photon, either of the nodes can be in the ionized state. This probability is measured by saving the CR-outcomes after the readout sequence. We include this ionized

state as a third level of our qubit, that is outside the space addressed by our microwave control. When applying a readout pulse however, it will be read out as the $|1\rangle$ (=dark) state. The single shot readout (SSRO) fidelity of the solid-state NV qubits are given in Table S3. State readout correction is performed according to iterative Bayesian unfolding as described in (*55*).

An overview of all the simulation parameters used and their measured/used value for the simulation is given in Table S4. We have also simulated near-term performance of the same link based on SnV centers (keeping the collection efficiency the same), which have 16 times higher coherent photon emission probability per optical excitation, full use of the improved QFC technology and improved phase stability.

For additional insight, we have simulated the infidelity contribution of the protocol error α and the SNR to the entangled state, as can be seen in Figure S11.

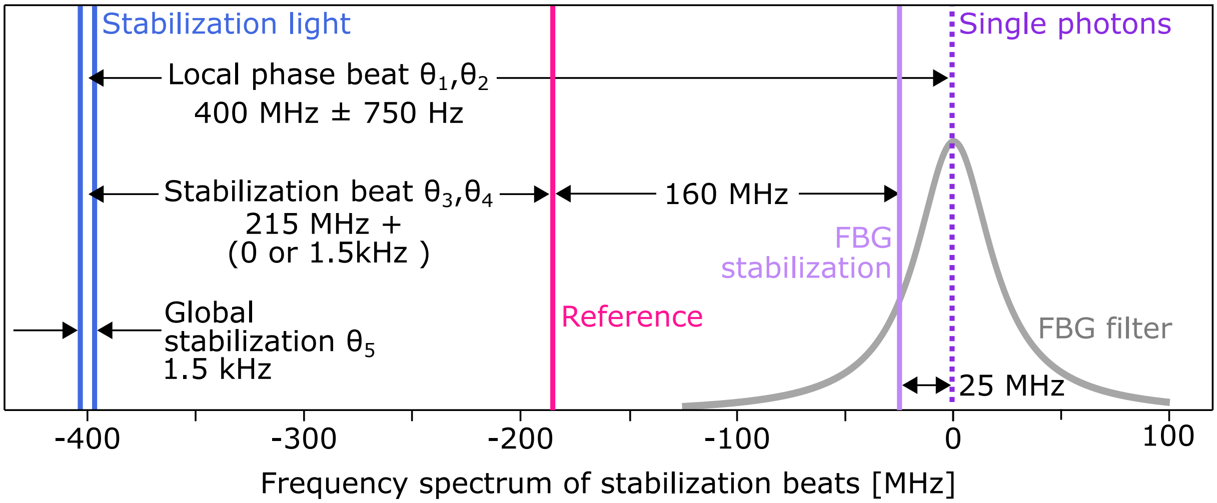

Figure S1: **Overview of beat frequencies and lay-out of optical fields used.** Frequency of relevant optical fields for the phase lock in the midpoint. The grey Lorentzian shape is the ultra-narrow fibre-bragg grating filter (FBG). It is stabilized using a $160\,\mathrm{MHz}$ beat generated by the reference laser and a frequency-shifted tap-off that is back-propagated through the flank of the FBG ($25\,\mathrm{MHz}$ from the transmission peak). The stabilization light is located far from the filter resonance, detuned by $400\,\mathrm{MHz}$ from the filter peak and excitation laser/single-photons, which is locked by the local phase lock controllers θ_1 and θ_2. This stabilization light is reflected off the FBG and interferes with the reference laser generating a $\approx 215\,\mathrm{MHz}$ beat, which is stabilized by the fast phase lock controller θ_3 and θ_4. The slight offset between the nodes of $1.5\,\mathrm{kHz}$ is measured by the SNSPDs and input to controller θ_5, to close the phase lock between Delft and The Hague.

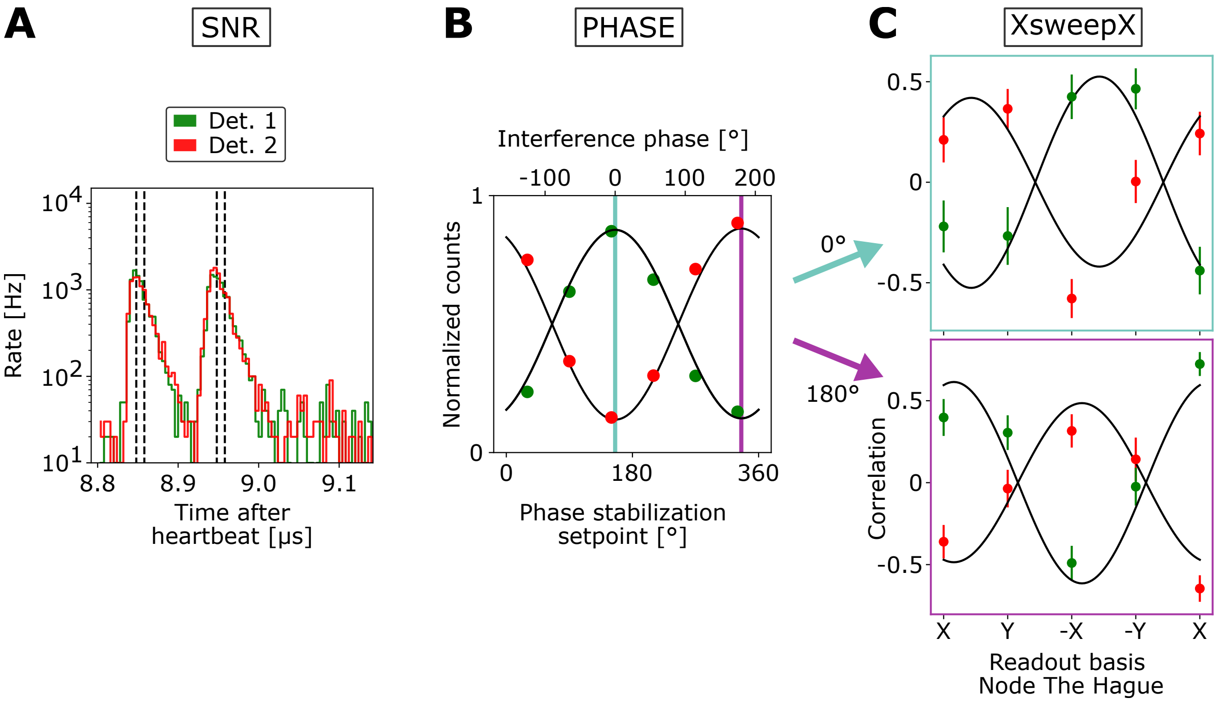

Figure S2: **Outcomes of calibration sequence to perform entanglement generation.** Typical calibration outcomes of: **(A)** signal strength (SNR) where the dotted lines show the used 'signal' window for both nodes respectively. The same windows are used to measure the 'noise' when the NV qubit state is rotated to the $|1\rangle$ (=dark) state.**(B)** Optical phase stability (PHASE), with fitted contrast (black lines). The vertical lines project to the top horizontal axis to determine the interference phase for the following measurements.**(C)** The entangled state phase (XsweepX) for an interference phase of $0°$ and $180°$, showing the capabilities of the stabilization system to set a specific phase s etpoint. The fitted phases (black solid lines) are 219(6) and 14(6) for the interference phase $0°$ and $180°$, respectively.

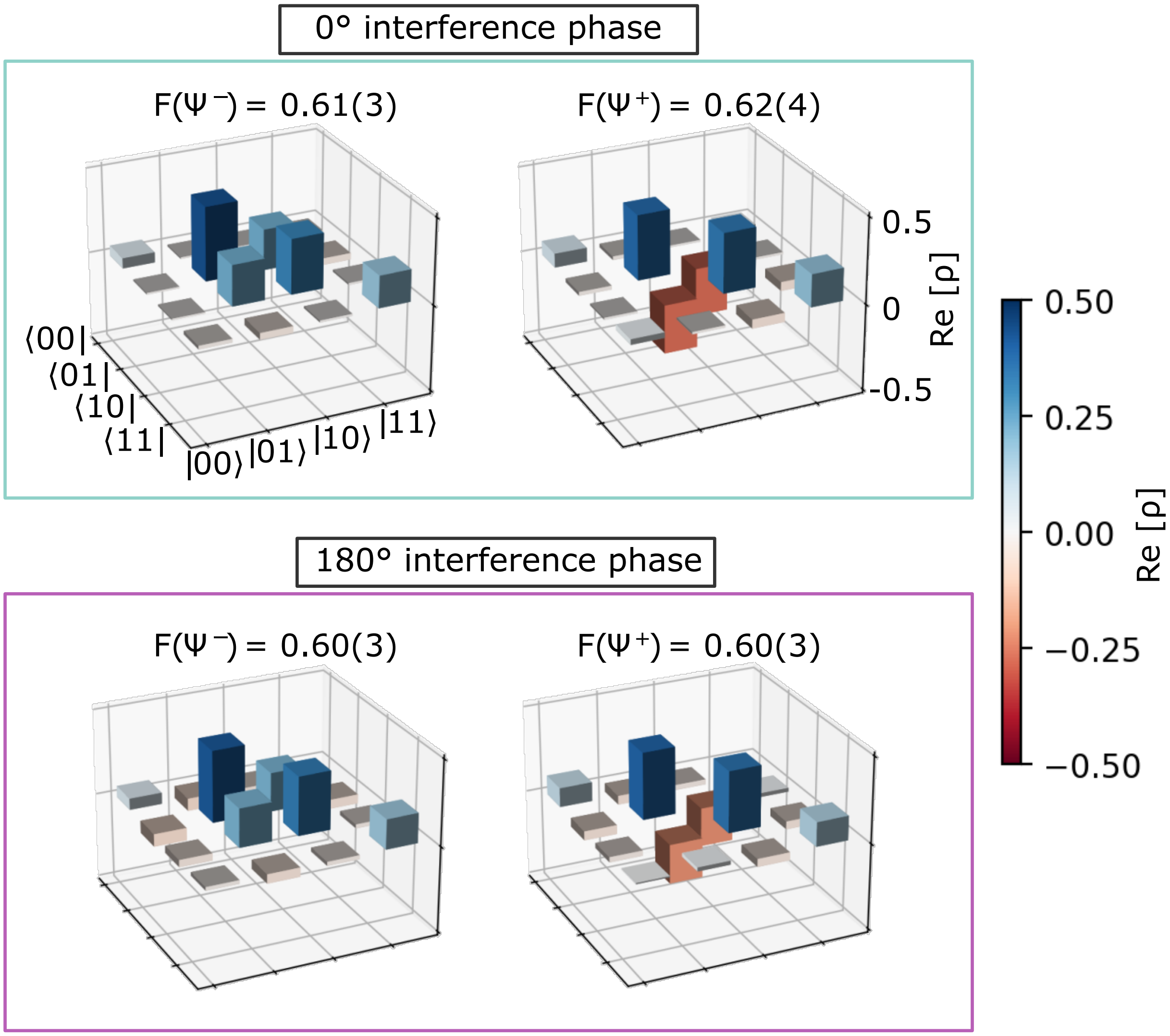

Figure S3: **Bayesian estimation of density matrix for both phase setpoints, using the post-selected entanglement generation scheme, with both nodes in the same lab in Delft.** We measure all two-spin correlators $\langle M_i M_j \rangle$ with $M_{i,j} \in [X, Y, Z]$ and use Bayesian estimation for tomography (*56, 57*) to find the most likely density matrix. The calibration measurements in Fig. S2 allow us to generate the same entangled state, at two different optical phase setpoints. The overlap with the ideal state for the most likely density matrix is given as numerical value, that is above 0.6 for both phase setpoints and detector outcomes.

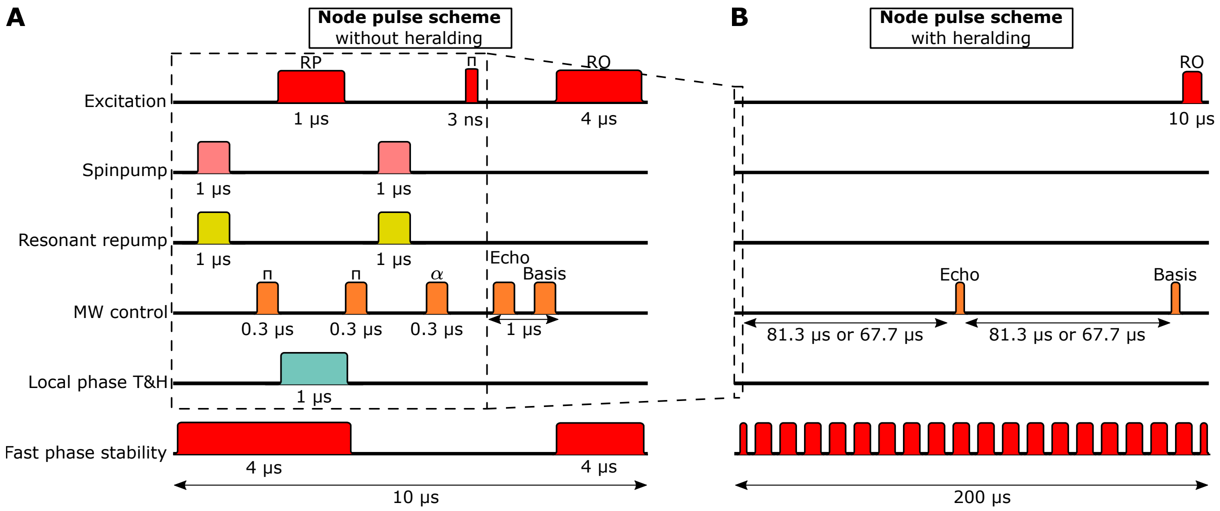

Figure S4: **Relative timing of control pulses on the nodes for entanglement generation without (A) and with (B) heralding.(A)** Local node pulse scheme for the duration of one heartbeat when performing entanglement generation without heralding. Red and yellow pulses are laser, orange are microwave pulses, blue-green is electrical feedback. The NV center is (re-)initialized in the correct spin and charge state via pumping resonant with $|1\rangle$ ($637\,\mathrm{nm}$) and the ZPL of NV^0($575\,\mathrm{nm}$), respectively. We limit resonant optical excitation during the reflection pulse (RP) we need for local phase stability by preparing the qubit state in the opposite spin state a using microwave π-pulse. After the local phase stability, we initialize the spin into the $|0\rangle$ state, create the unequal superposition with a microwave pulse with rotation angle α, and optically excite using a short optical excitation, completing the spin-photon entangled state generation. A microwave π-pulse echos the spin state, and a $\frac{\pi}{2}$-pulse selects the readout basis for the resonant optical readout that follows. **(B)** Node pulse scheme during entanglement generation. This sequence now takes 20 heartbeat periods ($200\,\mathrm{\mu s}$). All pulses required to perform the local phase stability and spin-photon entanglement are repeated exactly as in (A). However, now the rephasing echo pulse is played at its calibrated value of $82\,\mathrm{\mu s}$($68\,\mathrm{\mu s}$) for Node Delft (The Hague), while the stabilization light still is periodically sent to the midpoint such that phase stability feedback can be applied there. The total duration of $200\,\mathrm{\mu s}$ is for one whole entanglement generation attempt where wait time padding is added node specifically (not shown) to allow for a common repetition period when dealing with the different node-midpoint communication delays.

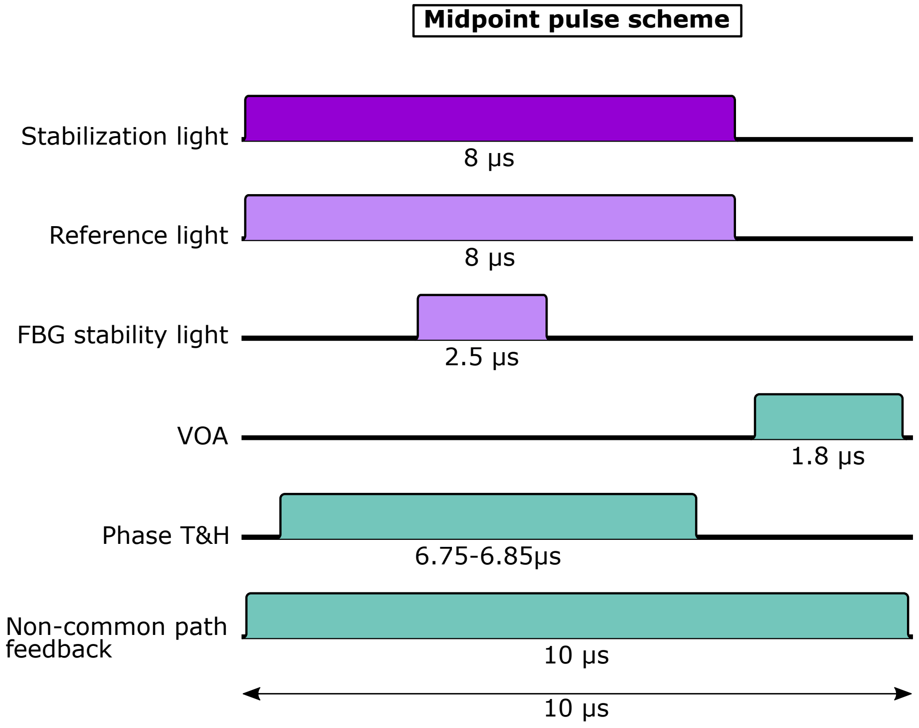

Figure S5: **Relative timing of midpoint pulse scheme for the duration of one heartbeat**. This sequence is agnostic to the experiment is run. Purple colors are telecom wavelength laser light, blue-green is electrical signal. Stabilization light is converted light coming from the nodes. The reference and FBG stability light are both originated from the telecom laser at the midpoint. The error signal for the fast lock is processed by a Track and Hold (T&H) amplifier before being processed by the controllers. Variable optical attenuators are used to shield the detectors during the times where stabilization/reference light is propagating in the system, and they are only opened for $2\,\mu s$ where single photons from the nodes can arrive. The global control task is not modulated, as the feedback bandwidth is much slower than the low-pass filtering of the error signal ($150\,\mathrm{Hz}$). For all above pulse schemes holds that absolute block sizes are not to scale, the denoted time duration is leading.

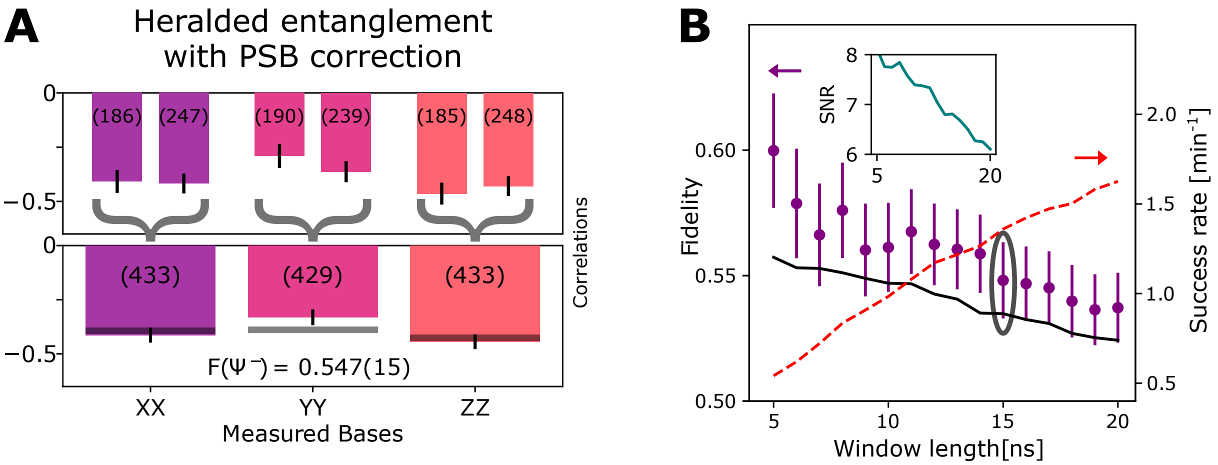

Figure S6: **Correlation measurement for full heralding with correction on discarding events accompanied by a measured PSB photon.** **(A)** Upper plot shows XX, YY and ZZ correlations per detector, lower for both detectors combined. Bars indicate measured data, the number in parenthesis indicate the amount of events. Horizontal lines indicate the theoretical model. The calculated fidelity is significantly above 0.5 **(B)** State fidelity and entanglement generation rate for varying photon acceptance window length. Inset shows signal-to-noise ratio for the same window lengths. All measurement outcomes are corrected for tomography errors, errorbars are 1 standard deviation.

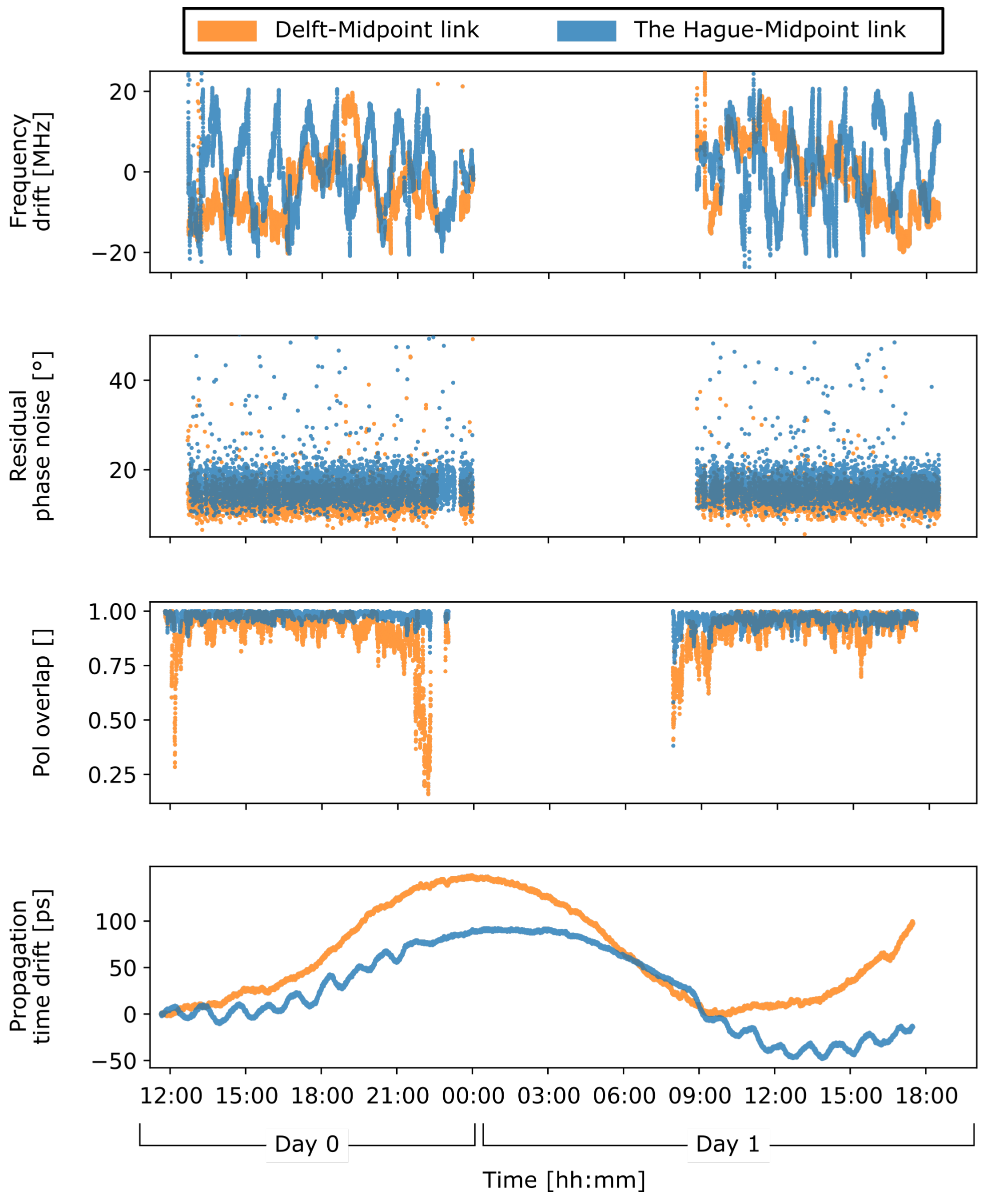

Figure S7: **Continuous data of important parameters monitored over deployed fiber link**. From top to bottom: the frequency drift, phase noise, polarization and time of flight as measured by the individual systems and logged into a central database. For the frequency, phase and polarization we have conditioned the data on the respective feedback system being active. The pause in the system overnight was due to one of the feedback systems being out of lock, which got reset the next morning.

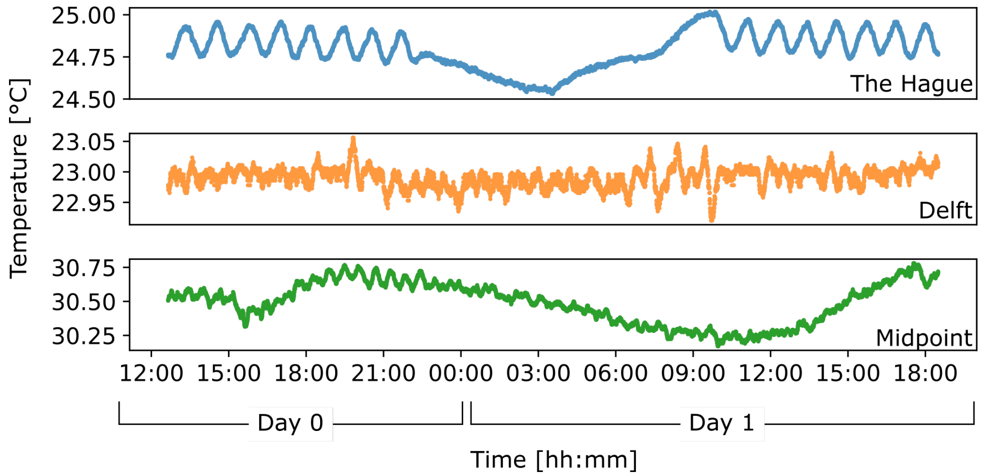

Figure S8: **Temperature logging of the three locations during the same time as the continuous data logging of Fig. S7.** Some of the features shown in S7, such as the frequency drifts, seem to be correlated with the measured temperature variations on the node.

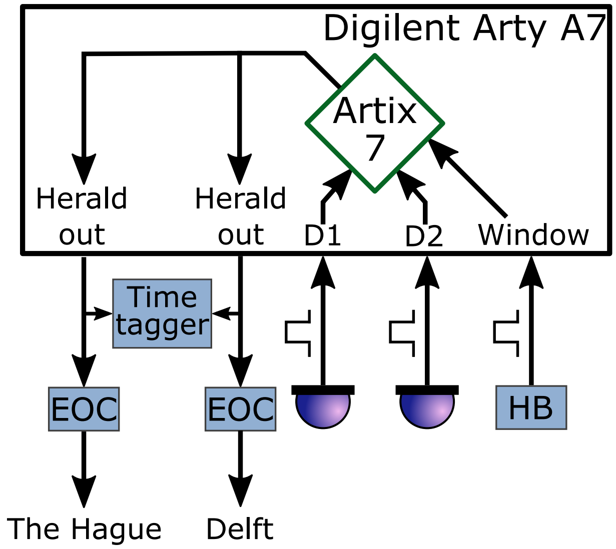

Figure S9: **Hardware lay-out of the FPGA used for heralding.** Single photon detection signals are processed by custom FPGA-code on the Digilent Arty A7. If photons arrive within the externally derived acceptance window from the heartbeat (HB), a heralding signal is sent to both locations through an Electrical-to-Optical-Converter (EOC). The heralding signal is also recorded on the timetaggers on all the three locations.

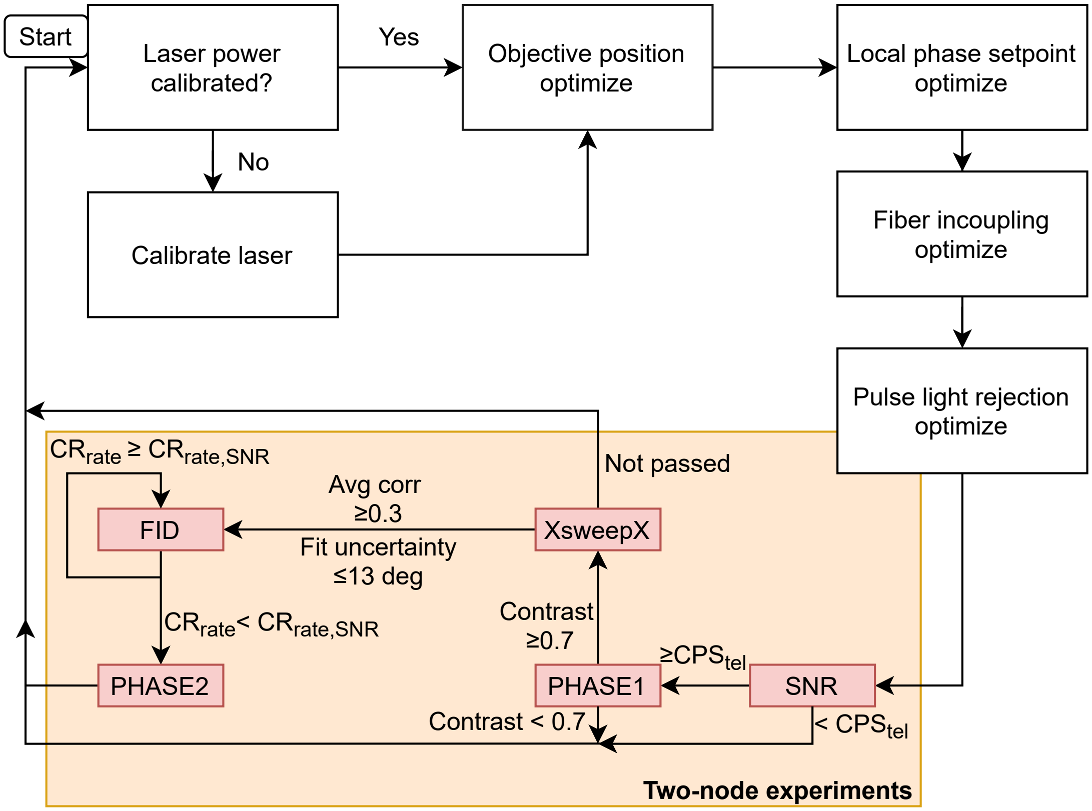

Figure S10: **Flowchart for calibrations and performing the two-node experiments of measuring the fidelity of the entangled state.** Between the different steps of the measurement sequence thresholds are checked of measured parameters that decide continuation of the sequence. Calibrations are started with a signal-to-noise (SNR) measurement, where the counts-per-shot of telecom signal from the nodes $(\mathrm{CPS}_{\mathrm{tel}})$ is checked. We continue with PHASE and XsweepX and check their respective parameters. If all thresholds are satisfied, we continue with the fidelity measurements (FID) until the Charge Resonance passing rate $(\mathrm{CR}_{\mathrm{rate}})$ drops below the calibrated value it had during the SNR $(\mathrm{CR}_{\mathrm{rate,SNR}})$. Finally, we do another PHASE check before we restart the entire cycle.

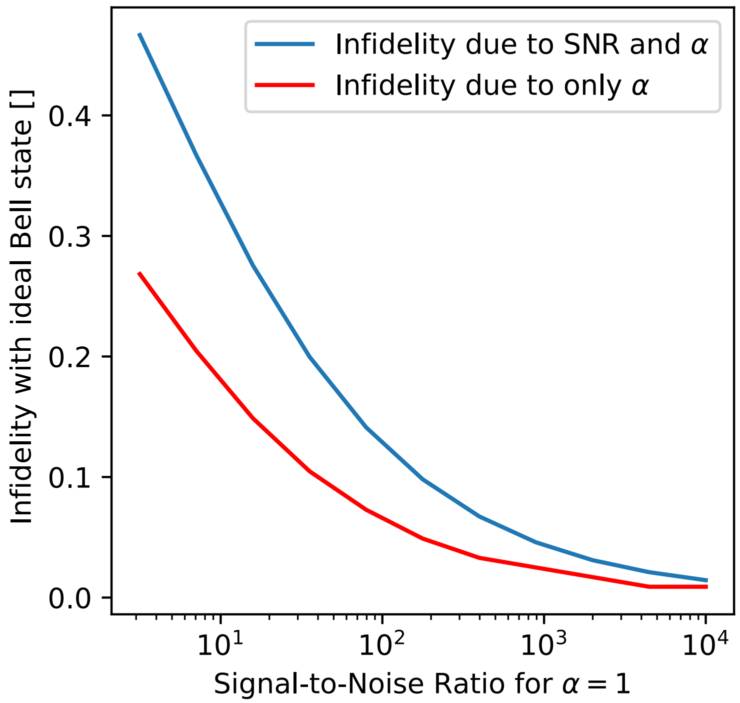

Figure S11: **Impact of the protocol error α on the entangled state fidelity for different signal-to-noise ratios (SNRs).** The red (bottom) line shows the infidelity contribution with respect to the ideal Bell state with only the protocol error α as contribution to this infidelity scaling as $1 - \alpha$. The blue line (top) is the infidelity contribution both due to the SNR and α, having a higher infidelity. The SNR is defined as , which is defined at $\alpha = 1$. Note that every point on both lines has an optimized α such that the infidelity contribution is the lowest, at the given SNR.

QFC	Node	Non-linear medium	Efficiency (front to end)	Noise $\left[s^{-1}\,GHz^{-1}\right]$	Eval. at filter bandwidth
QuTech (*22, 34*)	The Hague	ppLN waveguide	50%	2104	$50\,MHz$
Fraunhofer ILT (*35*)	Delft	bulk KTA	48%	19	$374\,GHz$

Table S1: **Comparison of the QFCs used in the experiment.**

Link	Loss local	Loss deployed fiber	Total	Typ. Round-trip time, drift
The Hague to midpoint	$3\,dB$ QFC, $4.9\,dB$ filters	$5.2\,dB$, $0.51\,dB\,km^{-1}$	$13.1\,dB$	$103.16\,\mu s$ $\pm 40\,ps\,h^{-1}$
Delft to midpoint	$4\,dB$ QFC, $4.5\,dB$ filters	$5.6\,dB$, $0.39\,dB\,km^{-1}$	$13.9\,dB$	$145.31\,\mu s$ $\pm 80\,ps\,h^{-1}$

Table S2: **Parameters of the losses and delays over the quantum channel.**

Table S3: **Error budget of deployed link post-selected and heralded entangled states.** Parameters noted with * are averages of data measured between the fidelity measurements, other are calibrated in separate experiments. The probability to detect a photon is given for $\alpha = 1$. If only one value is mentioned it is considered equal for both nodes. The infidelity contribution is given as if it was the only error present, to make direct comparison easier. Note that due to the large contribution of noise counts, the infidelity of the final simulated state with all the contributions taken into account does not follow the sum of the individual contributions.

Simulation parameter	Delayed-choice		Fully heralded	
	Value	Error con.	Value	Error con.
Det. prob.* (Delft, DH)[]	10.6e-6, 8.4e-6	28.1%	10.0e-6, 7.1e-6	26.5%
α []	0.25 and 0.25		0.25 and 0.25	
Backgr. counts* (Det1, Det2) [Hz]	40.3, 42.8		23.8, 22.0	
Double excitation probability []	0.04, 0.1	5.5%	0.04, 0.1	5.8%
Res. phase noise std* [°]	35	10.1%	41.1	12.3%
Dephasing probability []	0.01, 0.01	2.3%	0.02, 0.04	4.0%
Spectral diffusion FWHM [MHz]	25	6.5%	25	6.9%
Beam splitter imperfections []	0.95		0.95	
Detection window length [ns]	10		15	
Optical Excitation Rabi angle [°]	150, 150	-	150, 150	-
Ionization probability* []	0.035, 0.045%	5.4%	0.046	6.2%
PSB collection efficiency []	0.1, 0.1	-	0.0, 0.0	-
Simulated error (all params)	-	42.9%	-	45.8%
Simulated Fidelity	0.568, 0.576	-	0.544	-
Measured error	-	43.2%, 42.4%	-	46.6%
Avg. SSRO fidelity	95.14%, 94.49%	-	94.13%, 94.98%	

Table S4: **Error budget of near-term and future improvements.** For the near-term performance, we simulated an improved ZPL fraction of the SnV, the implementation of the improved QFC on both nodes, and an improvement of the local phase-stabilization. For future improvements on the system with we have added improvements in qubit coherence via dynamical decoupling, reduction of double excitation via shorter optical excitation,reduced ionization by better control of the charge state of the emitter and improved mode overlap at the central beamsplitter.

Simulation parameter	Near-term		Future	
	Value	Error con.	Value	Error con.
Det. prob. (Delft, DH)[]	16x10e-6, 16x10e-6	4.2%	16x10e-6, 16x10e-6	4.2%
α []	0.05 and 0.05		0.05 and 0.05	
Backgr. counts (Det1, Det2) [Hz]	5.0, 5.0		5.0, 5.0	
Double excitation probability []	0.04, 0.04	2.15%	0.01, 0.01	0.54%
Res. phase noise std [°]	15	1.7%	15	1.7%
Dephasing probability []	0.02, 0.04	3.0%	0.01, 0.01	1.0%
Spectral diffusion FWHM [MHz]	13	3.9%	13	1.3%
Beam splitter imperfections []	0.95		0.99	
Detection window length [ns]	15	-	15	-
Optical Excitation Rabi angle [°]	150, 150	-	150, 150	-
Ionization probability []	0.046	4.7%	0.01	1.0%
PSB collection efficiency []	0.1, 0.1	-	0.1, 0.1	-
Simulated error (all params)	-	18.9%	-	10.0%
Simulated Fidelity	0.811	-	0.90	-

REFERENCES AND NOTES

1. H. J. Kimble, The quantum internet. *Nature* **453**, 1023–1030 (2008).

2. S. Wehner, D. Elkouss, R. Hanson, Quantum internet: A vision for the road ahead. *Science* **362**, eaam9288 (2018).

3. H. Buhrman, R. Cleve, J. Watrous, R. de Wolf, Quantum fingerprinting. *Phys. Rev. Lett.* **87**, 167902 (2001).

4. M. Ben-Or, A. Hassidim, *Proceedings of the Thirty-Seventh Annual ACM Symposium on Theory of Computing , STOC '05* (Association for Computing Machinery, 2005), pp. 481–485.

5. D. Gottesman, T. Jennewein, S. Croke, Longer-baseline telescopes using quantum repeaters. *Phys. Rev. Lett.* **109**, 070503 (2012).

6. J. F. Clauser, M. A. Horne, A. Shimony, R. A. Holt, Proposed experiment to test local hidden-variable theories. *Phys. Rev. Lett.* **23**, 880–884 (1969).

7. D. L. Moehring, P. Maunz, S. Olmschenk, K. C. Younge, D. N. Matsukevich, L.-M. Duan, C. Monroe, Entanglement of single-atom quantum bits at a distance. *Nature* **449**, 68–71 (2007).

8. S. Ritter, C. Nölleke, C. Hahn, A. Reiserer, A. Neuzner, M. Uphoff, M. Mücke, E. Figueroa, J. Bochmann, G. Rempe, An elementary quantum network of single atoms in optical cavities. *Nature* **484**, 195–200 (2012).

9. J. Hofmann, M. Krug, N. Ortegel, L. Gérard, M. Weber, W. Rosenfeld, H. Weinfurter, Heralded entanglement between widely separated atoms. *Science* **337**, 72–75 (2012).

10. H. Bernien, B. Hensen, W. Pfaff, G. Koolstra, M. S. Blok, L. Robledo, T. H. Taminiau, M. Markham, D. J. Twitchen, L. Childress, R. Hanson, Heralded entanglement between solid-state qubits separated by three metres. *Nature* **497**, 86–90 (2013).

11. R. Stockill, M. J. Stanley, L. Huthmacher, E. Clarke, M. Hugues, A. J. Miller, C. Matthiesen, C. Le Gall, M. Atatüre, Phase-tuned entangled state generation between distant spin qubits. *Phys. Rev. Lett.* **119**, 010503 (2017).

12. L. Stephenson, D. Nadlinger, B. Nichol, S. An, P. Drmota, T. Ballance, K. Thirumalai, J. Goodwin, D. Lucas, C. Ballance, High-rate, high-fidelity entanglement of qubits across an elementary quantum network. *Phys. Rev. Lett.* **124**, 110501 (2020).

13. V. Krutyanskiy, M. Galli, V. Krcmarsky, S. Baier, D. Fioretto, Y. Pu, A. Mazloom, P. Sekatski, M. Canteri, M. Teller, J. Schupp, J. Bate, M. Meraner, N. Sangouard, B. Lanyon, T. Northup, Entanglement of trapped-ion qubits separated by 230 meters. *Phys. Rev. Lett.* **130**, 050803 (2023).

14. P. Drmota, D. Main, D. Nadlinger, B. Nichol, M. Weber, E. Ainley, A. Agrawal, R. Srinivas, G. Araneda, C. Ballance, D. Lucas, Robust quantum memory in a trapped-ion quantum network node. *Phys. Rev. Lett.* **130**, 090803 (2023).

15. M. Pompili, C. Delle Donne, I. te Raa, B. van der Vecht, M. Skrzypczyk, G. Ferreira, L. de Kluijver, A. J. Stolk, S. L. N. Hermans, P. Pawełczak, W. Kozlowski, R. Hanson, S. Wehner, Experimental demonstration of entanglement delivery using a quantum network stack. *npj Quantum Inf.* **8**, 121 (2022).

16. M. Pompili, S. L. N. Hermans, S. Baier, H. K. C. Beukers, P. C. Humphreys, R. N. Schouten, R. F. L. Vermeulen, M. J. Tiggelman, L. dos Santos Martins, B. Dirkse, S. Wehner, R. Hanson, Realization of a multinode quantum network of remote solid-state qubits. *Science* **372**, 259–264 (2021).

17. S. L. N. Hermans, M. Pompili, H. K. C. Beukers, S. Baier, J. Borregaard, R. Hanson, Qubit teleportation between non-neighbouring nodes in a quantum network. *Nature* **605**, 663–668 (2022).

18. P. Drmota, D. P. Nadlinger, D. Main, B. C. Nichol, E. M. Ainley, D. Leichtle, A. Mantri, E. Kashefi, R. Srinivas, G. Araneda, C. J. Ballance, D. M. Lucas, Verifiable blind quantum computing with trapped ions and single photons. *Phys. Rev. Lett.* **132**, 150604 (2024).

19. M. Bock, P. Eich, S. Kucera, M. Kreis, A. Lenhard, C. Becher, J. Eschner, High-fidelity entanglement between a trapped ion and a telecom photon via quantum frequency conversion. *Nat. Commun.* **9**, 1998 (2018).

20. V. Krutyanskiy, M. Meraner, J. Schupp, V. Krcmarsky, H. Hainzer, B. P. Lanyon, Light-matter entanglement over 50 km of optical fibre. *npj Quantum Inf.* **5**, 72 (2019).

21. M. Schäfer, B. Kambs, D. Herrmann, T. Bauer, C. Becher, Two-stage, low noise quantum frequency conversion of single photons from silicon-vacancy centers in diamond to the telecom c-band. *Adv. Quantum Technol.* 2300228 (2023).

22. A. Tchebotareva, S. L. Hermans, P. C. Humphreys, D. Voigt, P. J. Harmsma, L. K. Cheng, A. L. Verlaan, N. Dijkhuizen, W. de Jong, A. Dréau, R. Hanson, Entanglement between a diamond spin qubit and a photonic time-bin qubit at telecom wavelength. *Phys. Rev. Lett.* **123**, 063601 (2019).

23. E. Bersin, M. Sutula, Y. Q. Huan, A. Suleymanzade, D. R. Assumpcao, Y.-C. Wei, P.-J. Stas, C. M. Knaut, E. N. Knall, C. Langrock, N. Sinclair, R. Murphy, R. Riedinger, M. Yeh, C. Xin, S. Bandyopadhyay, D. D. Sukachev, B. Machielse, D. S. Levonian, M. K. Bhaskar, S. Hamilton, H. Park, M. Lončar, M. M. Fejer, P. B. Dixon, D. R. Englund, M. D. Lukin, Telecom networking with a diamond quantum memory. *PRX Quantum* **5**, 010303 (2024).

24. C. Bradley, J. Randall, M. Abobeih, R. Berrevoets, M. Degen, M. Bakker, M. Markham, D. Twitchen, T. Taminiau, A ten-qubit solid-state spin register with quantum memory up to one minute. *Phys. Rev. X* **9**, 031045 (2019).

25. V. Krutyanskiy, M. Canteri, M. Meraner, V. Krcmarsky, B. Lanyon, Multimode ion-photon entanglement over 101 kilometers. *PRX Quantum* **5**, 020308 (2024).

26. T. van Leent, M. Bock, F. Fertig, R. Garthoff, S. Eppelt, Y. Zhou, P. Malik, M. Seubert, T. Bauer, W. Rosenfeld, W. Zhang, C. Becher, H. Weinfurter, Entangling single atoms over 33 km telecom fibre. *Nature* **607**, 69–73 (2022).

27. C. M. Knaut, A. Suleymanzade, Y.-C. Wei, D. R. Assumpcao, P.-J. Stas, Y. Q. Huan, B. Machielse, E. N. Knall, M. Sutula, G. Baranes, N. Sinclair, C. De-Eknamkul, D. S. Levonian, M. K. Bhaskar, H. Park, M. Lončar, M. D. Lukin, Entanglement of nanophotonic quantum memory nodes in a telecom network. *Nature* **629**, 573–578 (2024).

28. D. Lago-Rivera, S. Grandi, J. V. Rakonjac, A. Seri, H. de Riedmatten, Telecom-heralded entanglement between multimode solid-state quantum memories. *Nature* **594**, 37–40 (2021).

29. X.-Y. Luo, Y. Yu, J.-L. Liu, M.-Y. Zheng, C.-Y. Wang, B. Wang, J. Li, X. Jiang, X.-P. Xie, Q. Zhang, X.-H. Bao, J.-W. Pan, Postselected entanglement between two atomic ensembles separated by 12.5 km. *Phys. Rev. Lett.* **129**, 050503 (2022).

30. J.-L. Liu, X.-Y. Luo, Y. Yu, C.-Y. Wang, B. Wang, Y. Hu, J. Li, M.-Y. Zheng, B. Yao, Z. Yan, D. Teng, J.-W. Jiang, X.-B. Liu, X.-P. Xie, J. Zhang, Q.-H. Mao, X. Jiang, Q. Zhang, X.-H. Bao, J.-W. Pan, Creation of memory-memory entanglement in a metropolitan quantum network. *Nature* **629**, 579–585 (2024).

31. D. Lago-Rivera, J. V. Rakonjac, S. Grandi, H. D. Riedmatten, Long distance multiplexed quantum teleportation from a telecom photon to a solid-state qubit. *Nat. Commun.* **14**, 1889 (2023).

32. C. Cabrillo, J. I. Cirac, P. Garcia-Fernández, P. Zoller, Creation of entangled states of distant atoms by interference. *Phys. Rev. A* **59**, 1025–1033 (1999).

33. S. Bose, P. L. Knight, M. B. Plenio, V. Vedral, Proposal for teleportation of an atomic state via cavity decay. *Phys. Rev. Lett.* **83**, 5158–5161 (1999).

34. A. J. Stolk, K. L. van der Enden, M.-C. Roehsner, A. Teepe, S. O. J. Faes, C. E. Bradley, S. Cadot, J. van Rantwijk, I. te Raa, R. A. J. Hagen, A. Verlaan, J. Biemond, A. Khorev, R. Vollmer, M. Markham, A. Edmonds, J. Morits, T. Taminiau, E. van Zwet, R. Hanson, Telecom-

band quantum interference of frequency-converted photons from remote detuned NV centers. *PRX Quantum* **3**, 020359 (2022).

35. J. F. Geus, F. Elsen, S. Nyga, A. J. Stolk, K. L. van der Enden, E. J. van Zwet, C. Haefner, R. Hanson, B. Jungbluth, Low-noise short-wavelength pumped frequency downconversion for quantum frequency converters. *Opt. Quantum* **2**, 189–195 (2024).

36. A. Dréau, A. Tchebotareva, A. E. Mahdaoui, C. Bonato, R. Hanson, Quantum frequency conversion of single photons from a nitrogen-vacancy center in diamond to telecommunication wavelengths. *Phys. Rev. Appl.* **9**, 064031 (2018).

37. S. L. N. Hermans, M. Pompili, L. D. S. Martins, A. R.-P. Montblanch, H. K. C. Beukers, S. Baier, J. Borregaard, R. Hanson, Entangling remote qubits using the single-photon protocol: An in-depth theoretical and experimental study. *New J. Phys.* **25**, 013011 (2023).

38. E. F. Dierikx, A. E. Wallin, T. Fordell, J. Myyry, P. Koponen, M. Merimaa, T. J. Pinkert, J. C. J. Koelemeij, H. Z. Peek, R. Smets, White rabbit precision time protocol on long-distance fiber links. *IEEE Trans. Ultrason. Ferroelectr. Freq. Control* **63**, 945–952 (2016).

39. C. A. Ryan, J. S. Hodges, D. G. Cory, Robust decoupling techniques to extend quantum coherence in diamond. *Phys. Rev. Lett.* **105**, 200402 (2010).

40. M. H. Abobeih, J. Cramer, M. A. Bakker, N. Kalb, M. Markham, D. J. Twitchen, T. H. Taminiau, One-second coherence for a single electron spin coupled to a multi-qubit nuclear-spin environment. *Nat. Commun.* **9**, 2552 (2018).

41. P. Lodahl, Quantum-dot based photonic quantum networks. *Quantum Sci. Technol.* **3**, 013001 (2018).

42. C. Bradac, W. Gao, J. Forneris, M. E. Trusheim, I. Aharonovich, Quantum nanophotonics with group IV defects in diamond. *Nat. Commun.* **10**, 5625 (2019).

43. N. T. Son, C. P. Anderson, A. Bourassa, K. C. Miao, C. Babin, M. Widmann, M. Niethammer, J. Ul Hassan, N. Morioka, I. G. Ivanov, F. Kaiser, J. Wrachtrup, D. D. Awschalom, Developing silicon carbide for quantum spintronics. *Appl. Phys. Lett.* **116**, 190501 (2020).

44. M. Ruf, N. H. Wan, H. Choi, D. Englund, R. Hanson, Quantum networks based on color centers in diamond. *J. Appl. Phys.* **130**, 070901 (2021).

45. J. V. Rakonjac, S. Grandi, S. Wengerowsky, D. Lago-Rivera, F. Appas, H. d. Riedmatten, Transmission of light-matter entanglement over a metropolitan network. *Opt. Quantum* **1**, 94–102 (2023).

46. M. Ruf, M. Weaver, S. van Dam, R. Hanson, Resonant excitation and purcell enhancement of coherent nitrogen-vacancy centers coupled to a fabry-perot microcavity. *Phys. Rev. Appl.* **15**, 024049 (2021).

47. D. Riedel, I. Söllner, B. J. Shields, S. Starosielec, P. Appel, E. Neu, P. Maletinsky, R. J. Warburton, Deterministic enhancement of coherent photon generation from a nitrogen-vacancy center in ultrapure diamond. *Phys. Rev. X* **7**, 031040 (2017).

48. A. Sipahigil, R. E. Evans, D. D. Sukachev, M. J. Burek, J. Borregaard, M. K. Bhaskar, C. T. Nguyen, J. L. Pacheco, H. A. Atikian, C. Meuwly, R. M. Camacho, F. Jelezko, E. Bielejec, H. Park, M. Lončar, M. D. Lukin, An integrated diamond nanophotonics platform for quantum-optical networks. *Science* **354**, 847–850 (2016).

49. M. Pasini, N. Codreanu, T. Turan, A. Riera Moral, C. F. Primavera, L. De Santis, H. K. C. Beukers, J. M. Brevoord, C. Waas, J. Borregaard, R. Hanson, Nonlinear quantum photonics with a tin-vacancy center coupled to a one-dimensional diamond waveguide. *Phys. Rev. Lett.* **133**, 023603 (2024).

50. P.-J. Stas, Y. Q. Huan, B. Machielse, E. N. Knall, A. Suleymanzade, B. Pingault, M. Sutula, S. W. Ding, C. M. Knaut, D. R. Assumpcao, Y.-C. Wei, M. K. Bhaskar, R. Riedinger, D. D. Sukachev, H. Park, M. Lončar, D. S. Levonian, M. D. Lukin, Robust multi-qubit quantum network node with integrated error detection. *Science* **378**, 557–560 (2022).

51. V. Krutyanskiy, M. Canteri, M. Meraner, J. Bate, V. Krcmarsky, J. Schupp, N. Sangouard, B. Lanyon, Telecom-wavelength quantum repeater node based on a trapped-ion processor. *Phys. Rev. Lett.* **130**, 213601 (2023).

52. N. Kalb, A. A. Reiserer, P. C. Humphreys, J. J. W. Bakermans, S. J. Kamerling, N. H. Nickerson, S. C. Benjamin, D. J. Twitchen, M. Markham, R. Hanson, Entanglement distillation between solid-state quantum network nodes. *Science* **356**, 928–932 (2017).

53. I. T. Raa, H. K. Ervasti, P. J. Botma, L. C. Visser, R. Budhrani, J. F. van Rantwijk, S. P. Cadot, J. Vermeltfoort, M. Pompili, A. J. Stolk, M. J. Weaver, K. L. van der Enden, D. de Leeuw Duarte, M. Teng, J. van Zwieten, F. Grooteman, QMI - quantum measurement infrastructure, a python 3 framework for controlling laboratory equipment (2023).

54. A. J. Stolk, J. J. B. Biemond, K. L. van der Enden, L. van Dooren, E. J. van Zwet, R. Hanson, Extendable optical phase synchronization of remote and independent quantum network nodes over deployed fibers. arXiv:2408.12464 [quant-ph] (2024).

55. M. Pompili, "Multi-node quantum networks with diamond qubits," thesis, Delft Univ. of Technology (2021).

56. C. Granade, J. Combes, D. G. Cory, Practical bayesian tomography. *New J. Phys.* **18**, 033024 (2016).

57. C. Granade, C. Ferrie, I. Hincks, S. Casagrande, T. Alexander, J. Gross, M. Kononenko, Y. Sanders, Qinfer: Statistical inference software for quantum applications. *Quantum* **1**, 5 (2017).
